# Perpetrator types, severity, and cumulative trauma: a population-based study of childhood sexual abuse characteristics and lifelong health

**DOI:** 10.1016/j.lanwpc.2026.101923

**Published:** 2026-07-13

**Authors:** Ben Mathews, Huyen P. Do, David Finkelhor, David M. Lawrence, James G. Scott, Daryl J. Higgins, Holly E. Erskine, Tracie O. Afifi, Jennie Noll

**Affiliations:** a School of Law, Queensland University of Technology (QUT), Brisbane, QLD, Australia; b Bloomberg School of Public Health, Johns Hopkins University, Baltimore, MD, USA; c Australian Centre for Health Law Research, Faculty of Business and Law, Queensland University of Technology, Brisbane, QLD, Australia; d Crimes against Children Research Center, Department of Sociology, University of New Hampshire, Durham, NH, USA; e School of Population Health, Curtin University, Perth, WA, Australia; f QIMR Berghofer, Medical Research Institute, Brisbane, QLD, Australia; g Queensland Centre for Mental Health Research, Wacol, QLD, Australia; h Child and Youth Mental Health Service, Children's Health QLD, South Brisbane, QLD, Australia; i Health Research Centre, The University of Queensland, Brisbane, QLD, Australia; j Institute of Child Protection Studies, Australian Catholic University, Melbourne, VIC, Australia; k School of Public Health, University of Queensland, Saint Lucia, QLD, Australia; l Institute for Health Metrics and Evaluation, University of Washington, Seattle, WA, USA; m Department of Psychiatry, University of Manitoba, Winnipeg, Manitoba, Canada; n Mt Hope Family Center, University of Rochester, Rochester, NY, USA

**Keywords:** Child sexual abuse, Child Abuse, Offenders or perpetrators - trends by perpetrator types, Adult, Child, Adolescent, Long-term outcomes, Child welfare, Australia/epidemiology, Mental, behavioural and physical health outcomes, Child protection and health policy

## Abstract

**Background:**

The influence of child sexual abuse (CSA) severity and perpetrator type on health problems in adulthood is poorly understood. We examined health outcomes associated with CSA according to severity, number of perpetrators, and perpetrator type.

**Methods:**

We analysed data from a nationally representative sample of 8503 participants. We assessed the influence of four levels of severity and eight types of perpetrators on mental disorders, health risk behaviours, and physical conditions, using multivariable models adjusted for confounders including other maltreatment.

**Findings:**

CSA was strongly associated with several mental disorders, health risk behaviours, and physical conditions. Forced intercourse presented highest odds of health problems compared to no CSA (e.g., cannabis dependence aOR 2.2, 95% CI 1.1–4.5; non-suicidal self-injury aOR 1.7, 1.2–2.5), and compared to other CSA types. CSA by multiple perpetrator types increased adverse outcomes, including non-suicidal self-injury (aOR 4.1, 3.0–5.5), and suicide attempt (aOR 3.8, 2.8–5.1). CSA by both adolescent and adult perpetrators was associated with the greatest risk for outcomes including non-suicidal self-injury (aOR 4.4, 3.0–6.4) and suicide attempt (aOR 3.4, 2.4–4.9) with equivalent adverse outcomes for individuals abused by adolescents only, and adults only. CSA by known adolescents, known adults, and unknown adults, elevated risk of health outcomes.

**Interpretation:**

Greater CSA severity and multiple perpetrator exposure elevates risk of health problems in adulthood. Both known and unknown perpetrators, and adult and adolescent perpetrators, increase risk. Results can inform clinical practice and policy in diverse sectors.

**Funding:**

National Health and Medical Research Council.

**Research in context**

**Evidence before this study**

We searched PubMed for articles published at any time from database inception until May 1, 2025, using the search terms “child∗ sexual abuse" [Title/Abstract] AND (“mental" [Title/Abstract] OR “health” [Title/Abstract]) AND (“life∗” [Title/Abstract] OR “adult∗” [Title/Abstract]) AND (“outcome∗” [Title/Abstract] OR “assoc∗” [Title/Abstract]) AND (“type” [Title/Abstract] OR “sever∗” [Title/Abstract] OR “perp∗” [Title/Abstract]). This search yielded 221 results. We also searched reference lists of known systematic reviews, meta-analyses and articles on the topic. In general, few studies employ conceptually sound approaches to the construct of CSA, the use of robust outcome measures (e.g., diagnostic instrumentation for mental disorders) is rare, and controlling for other forms of maltreatment is also rare. We identified fewer than ten studies that rigorously assessed health outcomes of child sexual abuse in adulthood, considering either abuse severity, or perpetrator type. No studies have comprehensively considered a broad range of associated health outcomes in adulthood, and different perpetrators, and different abuse severity, using a large nationally representative sample including a large subset who had experienced child sexual abuse.

**Added value of this study**

This study provides new evidence from a large nationally representative sample of Australians, revealing a clear relationship between CSA severity, and perpetrator profile (number and type), and multiple serious health problems in adulthood. To our knowledge, it is the first study to disentangle the effects of CSA severity, multiple perpetrator classes, perpetrator age (adolescent versus adult), and perpetrator relationship (known/unknown perpetrators), on a range of health problems in adulthood, adjusting for confounders including other child maltreatment (physical abuse, emotional abuse, neglect, exposure to domestic violence). Findings highlight markedly elevated risks of a range of health problems, including mental disorders, non-suicidal self-injury, and suicide attempt, posed by any CSA, severe types of CSA (e.g., forced intercourse), CSA experienced by multiple perpetrators, CSA by both adolescent and adult perpetrators, and CSA by known and unknown perpetrators. These novel findings about CSA risk profiles can inform prevention, intervention, and policy responses.

**Implications of all the available evidence**

CSA severity and multiple perpetrator exposure is associated with elevated risks of diverse adult health problems. CSA by both known and unknown perpetrators, and by both adult and adolescent perpetrators, present elevated risk of multiple outcomes. Public health strategies should prioritise prevention across the spectrum of CSA experiences. Expanded support services are essential to mitigate the long-term health consequences of CSA. Results can inform policy in health, child protection, education and legal sectors. Given its substantial prevalence and significant outcomes, investment in prevention of CSA is economically and socially justified, and targeted primary prevention should be accelerated including through comprehensive sexuality education. Health and education practitioners need to be able to provide support as necessary and at an early stage to those with lived experience of CSA, whether inflicted by adults or adolescents. Practitioners across sectors deserve continuing professional education to build awareness of these outcomes and facilitate enhanced care and prevention.

## Introduction

Childhood sexual abuse (CSA) involves contact and non-contact sexual acts by any person against a child aged under 18 who either cannot or does not consent, for the purpose of sexual gratification.^1^^,^^2^ CSA is a pervasive public health issue, with global prevalence of CSA involving physical contact estimated as 18.9% for females and 14.8% for males.^3^ Meta-analyses including non-contact CSA find higher prevalence than those limited to contact CSA only.^4^

A substantial body of evidence has demonstrated strong associations between CSA and adverse health outcomes. A review of meta-analyses found CSA was associated with higher odds of adult mental health problems (e.g., post-traumatic stress disorder (PTSD), depression, anxiety), risk behaviours (e.g., suicide attempts, non-suicidal self-injury (NSSI)), and physical conditions (e.g., obesity).^5^ A meta-analysis concluded CSA yielded adjusted odds ratios of 4.4 for PTSD, 2.2 for depressive disorders, 2.4 for anxiety disorders, and 2.0 for alcohol use disorders.^6^ A longitudinal study of individuals followed from birth to age 45 years found CSA survivors (n = 181) were more likely to experience internalising and externalising disorders, suicide attempts, health risk behaviours, and sexually transmitted diseases,7^7^ confirming other longitudinal research.^8^ A review of reviews concluded the relationship between survivor and perpetrator did not affect outcomes,^9^ although this was based on limited data.^10^ A twin study found severity of CSA, family perpetration, and multiple perpetrators is more strongly associated with depression and anxiety than less severe CSA or non-familial/single perpetrators.^11^

Theoretical frameworks have been proposed to explain the mechanisms through which CSA may lead to these outcomes. CSA has unique and multiple compounding mechanisms beyond other maltreatment types, which explain its capacity for amplified health problems.^12^ The broadly accepted traumagenic dynamics theory posits four core processes: traumatic sexualisation, betrayal, powerlessness, and stigmatisation.^13^ Consistent with this theory, it is plausible that CSA of greater severity (e.g., penetrative abuse) presents particularly high risk. Other frameworks such as betrayal trauma theory^14^ highlight that CSA by individuals who have close relational bonds with the victim (e.g., caregivers or family members) exacerbates deleterious outcomes due to shattered trust and attachment.^15^

Despite consensus linking CSA to adverse adult outcomes, limitations in existing studies present critical knowledge gaps.^5^ First, many studies rely on clinical or non-representative samples, constraining generalisability.^16^ Even the most rigorous recent population-based study investigating associations between CSA and the risk of 22 adverse outcomes across multiple life domains in adulthood^7^ had only 937 participants of whom only 181 reporting experiences of CSA. Second, notwithstanding some notable exceptions,^7^ few studies assess outcomes through life. Third, with rare exceptions,^8^ most studies assess outcomes of any CSA, without differentiating by type (such as penetrative vs. non-contact), frequency, or perpetrator relationship.^9^ Fourth, studies often employ limited definitions of CSA, which are not congruent with robust conceptual models.^1^^,^^2^ Fifth, few studies have controlled for exposure to other forms of child maltreatment, such as physical and emotional abuse, which are often co-occurring.^17^, ^18^, ^19^ Sixth, there is little large-scale research that disaggregates CSA by perpetrator type (including relationship to the child), number of perpetrators, or perpetrator age (adolescent vs. adult).^4^^,^^20^ Adolescent-perpetrated CSA, in particular, is rarely addressed despite adolescents representing a substantial proportion of CSA perpetrators,^20^ limiting understanding of their unique impact on survivors. Finally, the field is still developing an understanding of outcomes depending on whether the perpetrator is known or unknown to the child, despite theoretical relevance and potential implications for disclosure^21^ and intervention.^22^ A more nuanced understanding of these factors is essential to inform prevention and survivor support.

This study aimed to examine associations between CSA severity, number of perpetrators, and perpetrator type, and mental disorders, health risk behaviours, and physical conditions in adulthood. To our knowledge, it is the first study to disentangle the effects of CSA severity, multiple perpetrator classes, perpetrator age (adolescent versus adult), and perpetrator relationship (known/unknown perpetrators), on a range of health problems in adulthood, adjusting for confounders including other child maltreatment (physical abuse, emotional abuse, neglect, exposure to domestic violence). Informed by theory, we hypothesised that:

(H1) CSA is strongly associated with health problems in adulthood, with risk increasing with severity;

(H2) risk of health problems increases with multiple perpetrators;

(H3) risk of health problems is similar for those experiencing CSA by adults compared to adolescents;

(H4) risk of health problems is higher for those experiencing CSA by known perpetrators compared to unknown perpetrators.

## Methods

### Study design and data sources

We analysed data from the Australian Child Maltreatment Study (ACMS), a nationally representative survey of child maltreatment and associated outcomes in adulthood.^23^ The sample and methodology^24^ are detailed elsewhere. Briefly, 8503 individuals aged 16 years and older were recruited, between April and October 2021, using a mobile phone random digit dialling approach, which captured a sample representative of the population aged 16 years and over. Participants were informed that the study was about childhood experiences and health, and were invited to take part through a brief verbal introduction delivered at the time of contact. Survey weights were applied to align estimates with national census data. The target sample of 8500 respondents (3500 aged 16–24 years and 1000 in each of five older age groups) provided 80% power to estimate prevalence within one percentage point and detect gender of 2.6% points or temporal differences of about two percentage points.^25^ The sample was broadly representative of the Australian population by gender, Indigenous status, region, remoteness, and marital status, though participants were more likely to be Australian-born, more educated, and of higher socioeconomic status.^24^ Survey weights adjusted for selection probability and population differences.^24^ Non-response bias was minimal.^24^ Of 59,018 dialled numbers, 14.0% of contacted individuals participated (4.0% of total eligible after screening), standard for random digit dialling surveys with Census-calibrated weights achieving representativeness.

### Measures

#### Child maltreatment

We used the *Juvenile Victimisation Questionnaire–R2: Adapted Version (Australian Child Maltreatment Study)* to assess child maltreatment history. This instrument was modelled on the JVQ-R2, with carefully designed and validated adaptations to ensure accurate measurement.^26^ Following best practice in epidemiological surveys of child maltreatment,^25^ the ACMS assessed all five maltreatment types (physical, sexual, and emotional abuse, neglect, and exposure to domestic violence) up to age 18 years, using behaviourally-specific questions congruent with conceptual models.^27^ The ACMS assessed physical abuse by parents/caregivers (two items: moderate, severe); emotional abuse by parents (three items: hostility, rejection, emotional unavailability); neglect (three items: environmental, nutritional/physical, medical); and exposure to domestic violence between parents (four items: assault, threatened assault, property damage, coercive control).

#### Child sexual abuse

**Fig. 1:**
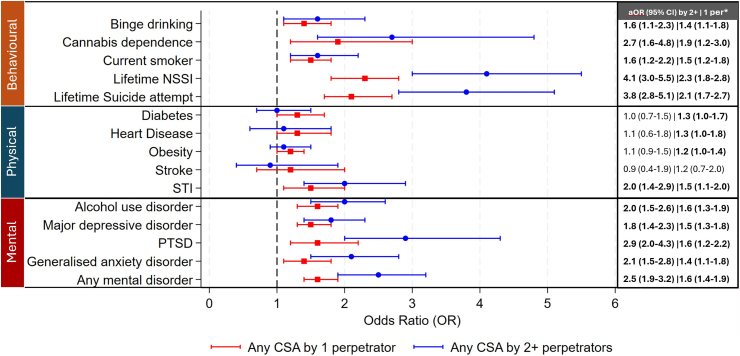
**Associations between childhood sexual abuse and adult outcomes by number of perpetrator classes.** (∗) Fully Adjusted Odds Ratios (95% CI) are shown for any CSA by 2+ perpetrator classes vs. No CSA and by 1 perpetrator class vs. No CSA. Models are fully adjusted for age, gender, experience of financial hardship during childhood, current financial strain, and other types of child maltreatment (physical abuse, emotional abuse, neglect, and exposure to domestic violence). Bold values indicate p < 0.05.

We used the comprehensive conceptual framework of CSA developed by Mathews and Collin-Vézina (2019), within which CSA includes both contact and non-contact sexual acts, experienced before age 18, inflicted by any person, where the act is for sexual gratification, and the child either cannot or does not consent.^1^ Informed by this model, four items assessed experiences of CSA inflicted by any individual.^1^^,^^2^ These comprised: (i) non-contact CSA (“Did anyone ever look at your private parts when they shouldn't have, or make you look at their private parts?”); (ii) sexual touching (“Did anyone ever touch your private parts when they shouldn't have, or make you touch their private parts?”); (iii) attempted forced intercourse (“Did anyone ever try to force you to have sex, even if it didn't happen?”); and (iv) completed forced intercourse (“Did anyone ever force you to have sex?”). Interviewers also clarified that the questions on attempted forced intercourse and forced intercourse referred to vaginal, oral, or anal intercourse involving any body part or object. For each type experienced, we asked about the perpetrator's identity and grouped responses into eight perpetrator classes: four involving known or unknown adults (parents/adult family members; institutional caregivers e.g., teachers; other known adults; unknown adults), and four involving known or unknown adolescents aged under 18 years (siblings; current or former romantic partners; other known adolescents; unknown adolescents). We use the term perpetrator classes to refer to eight collapsed groups of perpetrator types, consistent with our previous analysis.^20^ Experiences of CSA across perpetrator classes were not mutually exclusive as some participants experienced CSA by multiple perpetrators. Fig. 1 shows the participant flow chart. Among 2348 participants (28.5%) reporting any CSA, 1640 (69.8%) experienced CSA by perpetrators from a single class, and 708 (30.2%) by multiple classes. To avoid confounding from disclosures related to multiple perpetrator types, analyses of research hypothesis 4 were restricted to those reporting CSA by a single perpetrator class (n = 1640).^20^ Table S4 provides full details.

### Outcome measures

#### Mental, physical and behavioural outcomes

We used a diagnostic instrument to assess mental disorders and validated instrumentation for other outcomes. Behavioural outcomes included lifetime NSSI and suicide attempt with items from the National Adolescent Mental Health Survey,^28^ other health risk behaviours (smoking, binge drinking), and severe cannabis dependence using the Severity of Dependence Scale with cut-off point ≥ 3.^29^ Mental health outcomes were assessed using the Mini International Neuropsychiatric Interview (MINI), version 7.0.2^30^ to capture clinically diagnostic data on four disorders having established associations with CSA^31^: Generalised Anxiety Disorder (GAD) (current), Post-traumatic Stress Disorder (PTSD) (current), Alcohol Use Disorder (AUD) (current), and Major Depressive Disorder (MDD) (lifetime).^32^ Physical outcomes (diabetes, stroke, heart disease, sexually transmitted infections (STI)) were measured using items from the National Survey of Mental Health and Wellbeing (2007),^33^ and obesity (BMI ≥30 kg/m^2^) was derived from self-reported height and weight.^34^ Sexually transmitted infection (STI) history was assessed with the question: “Have you ever been told by a doctor or nurse that you have a sexually transmitted infection?”.

#### Covariates

The ACMS collected extensive information on demographics including age, gender, country of birth, educational level, residential socio-economic status, Indigenous status, marital status, and current financial strain. We controlled for the potential confounders including age groups (16–24, 25–44, 45+ years), gender, childhood familial financial hardship (difficulty buying food, medical care, or necessities), financial strain (past 12 months), socio-economic status (using Index of Relative Socio-Economic Disadvantage quintiles) and other child maltreatment types (physical abuse, emotional abuse, neglect, and exposure to domestic violence).

### Statistical analysis

Analyses were conducted in SAS 9.4, with graphs and plots generated in Stata 17. A two-sided p-value <0.05 indicated statistical significance.

#### Prevalence estimation

To ensure our sample reflected the Australian population, survey data were weighted by age group, gender, Indigenous status, country of birth (Australia or overseas), highest educational attainment, and residential socio-economic status (Relative Socio-economic Advantage and Disadvantage quintiles). We then estimated the survey-weighted prevalence of each health outcome across all CSA exposure groups (no CSA, any CSA, the four CSA subtypes, CSA by adult or adolescent perpetrators, and CSA by known or unknown adult or adolescent perpetrators). The Rao–Scott χ^2^ test with second-order correction assessed differences in prevalence. 95% Confidence Intervals (CIs) were estimated using Taylor series linearisation.^35^

#### Primary analysis

Multivariable logistic regression models were fitted using PROC SURVEYLOGISTIC to calculate adjusted odds ratios (aORs) for associations between CSA and health outcomes. For each outcome, six models were fitted to examine different dimensions of CSA experiences: **Model 1**: Any CSA vs no CSA (overall CSA impact); **Model 2**: Each subtype (non-contact, touching, attempted intercourse, completed intercourse) vs all without that subtype (including no CSA + other CSA types), estimates subtype-specific effects accounting for co-occurrence; **Model 3**: Completed/attempted intercourse vs all other CSA types (not no-CSA)-tests if most severe forms confer additional risk beyond other CSA, avoiding no-CSA dilution; **Models 4**–**6**: Perpetrator characteristics (number of classes, age group, familiarity) vs no CSA. This design tests both overall CSA impact and within-CSA heterogeneity by severity and perpetrator characteristics.

The simple model adjusted for age and gender. The fully adjusted model additionally controlled for childhood financial hardship, current financial strain, other maltreatment (physical abuse, emotional abuse, neglect, exposure to domestic violence). Socio-economic status was included in Models 1 and 2. No formal multiple comparisons correction was applied a priori, given the conceptually related nature of the outcomes; interpretation therefore focuses on patterns and effect sizes rather than isolated p-values.

#### Secondary analysis (pairwise comparisons)

To evaluate whether CSA by both adolescents and adults or known perpetrators posed higher risks than single or unknown perpetrators, pairwise contrasts were performed using ESTIMATE statements in logistic regression model 5 and 6. Model 5 compared OR from: (1) both adolescent and adult perpetrators vs adolescent-only perpetrators; (2) both vs adult-only perpetrators; (3) adult-only vs adolescent-only perpetrators. Model 6 compared OR from: (1) any known vs any unknown perpetrator; (2) any known adult vs any known adolescent; (3) any known adolescent vs any unknown adolescent; (4) any known adult vs any unknown adult. To ensure mutually exclusive groups and avoid overlap across perpetrator classes, individuals with CSA by perpetrators from more than one comparison category were excluded from these analyses. Contrast vectors (e.g., [0, 1, −1, 0]) tested differences in log-odds. Adjusted ORs with 95% CIs were exponentiated. Likelihood Ratio Test (omnibus test) confirmed absence of Type 1 errors from multiple comparisons (Table S6).

#### Sensitivity analyses

We computed E-values for statistically significant fully adjusted associations using the standard formula for odds ratios converted to risk ratios where appropriate. Larger E-values therefore indicate that substantial unmeasured confounding would be required to negate the observed effect.^36^

#### Model diagnostics

Model fit was assessed using Likelihood Ratio Tests and Goodness-of-Fit tests. Multicollinearity was ruled out via Variance Inflation Factors (VIFs) < 2 across models. No meaningful violations of linearity (logistic regression standard) or interaction effects were detected. Minimal missingness (CSA screeners 0.8–0.9%; perpetrator type <0.7%) justified complete-case analysis without imputation, appropriate for low missingness under missing at random assumption, unlikely to bias estimates.

### Ethics approval

Ethics approval was granted by the Queensland University of Technology Human Research Ethics Committee on 16 August 2019 (ID#1900000477). All participants provided verbal informed consent prior to participation.

### Role of the funding source

National Health and Medical Research Council had no role in study design, data collection, analysis, interpretation, or writing of this report.

## Results

### Severity: prevalence of health outcomes by type of CSA

Across the entire sample of 8503 participants, one in four (28.5%, n = 2348) experienced CSA, with higher prevalence among women, and little difference by age group. Individuals with CSA had significantly higher prevalence of all health risk behaviours, all mental disorders, and two physical health conditions (obesity, STI), compared to those without CSA (Table 1).

**Table 1:** Prevalence estimates of health outcomes by CSA exposure and type (n = 8503).

Characteristic and outcomes	No CSA		Any CSA		Non-contact CSA		Contact CSA					
					Exposure or voyeurism		Touching		Attempted forced intercourse		Completed forced intercourse	
	n	% (95% CI)	n	% (95% CI)	n	% (95% CI)	n	% (95% CI)	n	% (95% CI)	n	% (95% CI)
Gender	6155	71.5 (70.2–72.7)	2348	28.5 (27.3–29.8)	1443	18.1 (17.0–19.1)	1525	18.9 (17.9–20.0)	1201	13.8 (12.9–14.7)	717	8.7 (7.9–9.4)
Women (n = 4182)	2646	62.7 (60.8–64.5)	1536	37.3 (35.5–39.2)	911	22.9 (21.3–24.5)	1017	25.5 (23.8–27.2)	861	19.4 (17.9–21.0)	519	12.5 (11.2–13.8)
Men (n = 4195)	3456	81.2 (79.7–82.7)	739	18.8 (17.3–20.3)	476	12.6 (11.3–13.9)	452	11.6 (10.4–12.9)	296	7.4 (6.4–8.5)	167	4.3 (3.5–5.2)
Diverse genders (n = 126)	53	48.1 (35.4–60.7)	73	52 (39.3–65)	56	38.6 (26.4–50.7)	56	41.0 (28.7–53.3)	44	31.7 (20.3–43.0)	31	23.4 (13.1–33.6)
Age Group	6155	71.5 (70.2–72.7)	2348	28.5 (27.3–29.8)	1443	18.1 (17.0–19.1)	1525	18.9 (17.9–20.0)	1201	13.8 (12.9–14.7)	717	8.7 (7.9–9.4)
16–24 years (n = 3500)	2611	74.3 (72.7–75.9)	889	25.7 (24.1–27.3)	506	15.0 (13.7–16.4)	555	16.2 (14.9–17.6)	525	15.2 (13.9–16.5)	303	8.7 (7.7–9.7)
25–44 years (n = 2000)	1432	71.2 (69.0–73.4)	568	28.8 (26.6–31.0)	367	18.6 (16.7–20.5)	375	19.2 (17.3–21.1)	252	13.4 (11.7–15.1)	163	9.1 (7.6–10.5)
45 years or more (n = 3003)	2112	70.9 (69.1–72.7)	891	29.1 (27.3–30.9)	570	18.6 (17.0–20.1)	595	19.5 (17.9–21.1)	424	13.7 (12.3–15.0)	251	8.4 (7.3–9.5)
Health risk behaviours												
Smoking (n = 1312)	798	14.8 (13.6–16.0)	514	23.5 (21.3–25.7)	323	23.9 (21.1–26.7)	345	23.9 (21.2–26.6)	309	28.7 (25.3–32.1)	209	31.1 (26.6–35.5)
Binge drinking (n = 868)	590	10.2 (9.3–11.2)	278	13.0 (11.3–14.8)	172	14.1 (11.7–16.4)	177	12.7 (10.6–14.8)	160	13.6 (11.1–16.0)	98	14.8 (11.3–18.2)
Cannabis dependence (n = 259)	129	1.7 (1.3–2.1)	130	4.6 (3.5–5.6)	94	5.4 (4.0–6.8)	90	4.7 (3.4–5.9)	91	6.0 (4.4–7.7)	67	7.6 (5.2–10.0)
Lifetime NSSI (n = 1676)	803	9.6 (8.7–10.5)	873	29.4 (27.1–31.6)	553	31.9 (28.9–34.9)	612	31.8 (28.9–34.6)	547	35.7 (32.3–39.1)	368	41.8 (37.2–46.4)
Lifetime suicide attempt (n = 948)	401	5.9 (5.2–6.7)	547	21.8 (19.7–23.9)	368	23.9 (21.2–26.7)	408	24.4 (21.7–27.0)	376	28.9 (25.6–32.1)	275	34.1 (29.7–38.4)
Mental disorders												
Any mental disorder (n = 3606)	2221	31.8 (30.3–33.3)	1385	53.6 (51.0–56.1)	881	56.0 (52.7–59.2)	914	55.1 (52.0–58.2)	809	61.8 (58.2–65.4)	512	68.2 (63.8–72.7)
Alcohol use disorder (n = 1888)	1210	17.1 (15.9–18.3)	678	25.3 (23.1–27.5)	414	25.5 (22.7–28.2)	438	24.5 (21.9–27.2)	387	28.3 (25.1–31.5)	232	30.5 (26.2–34.8)
PTSD (n = 488)	183	3.1 (2.6–3.7)	305	10.8 (9.3–12.4)	226	12.5 (10.6–14.5)	236	12.3 (10.3–14.2)	228	16.3 (13.7–18.8)	165	18.9 (15.5–22.3)
Generalised anxiety disorder (n = 1148)	596	8.3 (7.4–9.2)	552	20.1 (18.1–22.1)	369	22.3 (19.7–24.9)	391	22.5 (19.9–25.0)	342	25.1 (22.1–28.2)	235	29.9 (25.6–34.1)
Major depressive disorder (n = 1716)	1010	14.3 (13.3–15.4)	706	28.5 (26.2–30.8)	439	29.1 (26.2–32.0)	459	29.4 (26.6–32.3)	400	31.9 (28.6–35.3)	257	35.9 (31.4–40.4)
Physical health conditions												
Obesity (n = 1794)	1189	24.9 (23.5–26.4)	605	31.4 (28.9–33.8)	385	30.1 (27.1–33.1)	424	34.3 (31.2–37.4)	326	35.1 (31.4–38.7)	204	36.1 (31.4–40.8)
Diabetes (n = 583)	397	9.1 (8.1–10.0)	186	11.6 (9.8–13.4)	124	12.1 (9.8–14.3)	132	12.3 (10.1–14.5)	86	10.5 (8.1–12.9)	46	9.9 (6.9–13.0)
Stroke (n = 131)	90	2.3 (1.8–2.8)	41	2.7 (1.8–3.6)	30	3.3 (2.0–4.6)	24	2.4 (1.4–3.4)	29	3.8 (2.3–5.3)	20	4.4 (2.3–6.5)
Heart disease (n = 412)	287	7.0 (6.1–7.9)	125	8.0 (6.5–9.6)	73	7.2 (5.4–9.0)	82	8.4 (6.4–10.4)	66	8.3 (6.1–10.4)	36	7.6 (4.8–10.4)
STI (n = 588)	314	5.5 (4.8–6.3)	274	11.0 (9.4–12.6)	174	11.2 (9.3–13.1)	183	11.8 (9.8–13.8)	178	13.7 (11.2–16.1)	128	16.1 (12.8–19.4)

Percentages are weighted with 95% confidence intervals. Sub-totals for each CSA type add to more than n = 2348 due to some participants experiencing more than one type of CSA.

Among all who experienced any CSA, 29.4% had experienced lifetime NSSI, 21.8% experienced lifetime suicide attempt, 28.5% had experienced lifetime major depressive disorder, and 11% had contracted a sexually transmitted infection. Among those who experienced completed forced intercourse (8.7% prevalence among the entire population), the weighted prevalences of these outcomes were 41.8% (NSSI), 34.1% (suicide attempt), 35.9% (MDD), and 16.1% (STI), respectively.

A clear increase in risk was presented by more severe CSA types, although the first two exposure groups were similar for some outcomes and the pattern was not strictly monotonic across all four categories. For example, lifetime suicide attempt was 5.9% among individuals without CSA, compared to 21.8% (any CSA), and 34.1% (forced intercourse). Table S1, S2, S9 provides full details.

### Severity: each type of CSA

Table 2 indicates associations between each type of CSA and health outcomes via both simply adjusted and fully adjusted logistic regression models, using “no CSA” as the reference group assess the overall and subtype-specific effects of CSA. Odds ratios indicate increasing risk according to abuse severity. More severe CSA - e.g., completed and attempted forced intercourse presented higher odds of health problems, even after adjusting for confounders (gender, age, other types of child maltreatment, financial hardship). Forced intercourse presented higher odds of cannabis dependence: OR 2.2 (1.1–4.5), lifetime NSSI: OR 1.7 (1.2–2.5), suicide attempts: OR 1.6 (1.1–2.3), and any mental disorder: OR 1.8 (1.3–2.5), major depressive disorder: OR 1.4 (1.1–1.9), STI: OR 1.5 (1.1–2.3). Less severe CSA (non-contact and touching) typically showed weaker or non-significant or non-significant associations after full adjustment. Details and full results of E-value estimates are provided in Table S10.

**Table 2:** Association between different subtypes of childhood sexual abuse and adult health problems (n = 8503).

Outcomes	Model 1: Any CSA vs No CSA		Model 2: CSA subtypes vs. no exposure to that subtype including those with no CSA exposure^a^							
			Non-contact CSA vs. no non-contact CSA		Touching vs. no touching		Attempted forced intercourse vs. no attempted forced intercourse		Completed forced intercourse vs. no completed forced intercourse	
	Simple adjustment odds ratio (95% CI)^b^	Fully adjusted odds ratio (95% CI)^c^	Simple adjustment odds ratio (95% CI)^b^	Fully adjusted odds ratio (95% CI)^c^	Simple adjustment odds ratio (95% CI)^b^	Fully adjusted odds ratio (95% CI)^c^	Simple adjustment odds ratio (95% CI)^b^	Fully adjusted odds ratio (95% CI)^c^	Simple adjustment odds ratio (95% CI)^b^	Fully adjusted odds ratio (95% CI)^c^
Health risk behaviours										
Current smoker (n = 1312)	2.0 (1.7–2.3) ∗∗∗	1.5 (1.3–1.8) ∗∗∗	1.2 (0.9–1.5)	1.1 (0.9–1.4)	1.1 (0.9–1.4)	1.0 (0.8–1.3)	1.8 (1.3–2.4) ∗∗∗	1.5 (1.1–2.1) ∗∗	1.4 (1.0–2.0) ∗	1.3 (0.9–1.9)
Binge drinking (n = 868)	1.5 (1.3–1.9) ∗∗∗	1.4 (1.2–1.8) ∗∗∗	1.4 (1.1–1.9) ∗	1.4 (1.1–1.8) ∗	1.0 (0.7–1.4)	1.0 (0.7–1.3)	1.3 (0.8–1.8)	1.1 (0.8–1.7)	1.3 (0.8–2.1)	1.3 (0.8–2.0)
Cannabis dependence (n = 259)	3.5 (2.5–5.1) ∗∗∗	2.2 (1.4–3.4) ∗∗∗	2.2 (1.3–3.7) ∗∗	1.9 (1.1–3.1) ∗	1.0 (0.5–1.8)	0.8 (0.4–1.5)	1.7 (0.9–3.5)	1.2 (0.6–2.5)	2.2 (1.1–4.2) ∗	2.2 (1.1–4.5) ∗
Lifetime NSSI (n = 1676)	4.2 (3.6–5.0) ∗∗∗	2.8 (2.3–3.3) ∗∗∗	2.0 (1.6–2.6) ∗∗∗	1.7 (1.3–2.2) ∗∗∗	1.7 (1.3–2.2) ∗∗∗	1.4 (1.1–1.8) ∗∗	1.6 (1.2–2.2) ∗∗	1.3 (1.0–1.8)	1.9 (1.3–2.6) ∗∗∗	1.7 (1.2–2.5) ∗∗
Lifetime suicide attempt (n = 948)	4.4 (3.6–5.3) ∗∗∗	2.7 (2.2–3.3) ∗∗∗	1.8 (1.3–2.3) ∗∗∗	1.5 (1.1–2.0) ∗	1.7 (1.2–2.3) ∗∗∗	1.3 (1.0–1.8)	2.0 (1.4–2.8) ∗∗∗	1.5 (1.1–2.1) ∗∗	1.8 (1.3–2.5) ∗∗∗	1.6 (1.1–2.3) ∗∗
Mental disorders										
Any mental disorder (n = 3606)	2.7 (2.4–3.1) ∗∗∗	1.8 (1.6–2.1) ∗∗∗	1.6 (1.3–1.9) ∗∗∗	1.3 (1.1–1.6) ∗∗	1.3 (1.1–1.6) ∗∗	1.1 (0.9–1.3)	1.7 (1.4–2.2) ∗∗∗	1.4 (1.1–1.8) ∗	1.9 (1.4–2.5) ∗∗∗	1.8 (1.3–2.5) ∗∗∗
Alcohol use disorder (n = 1888)	2.0 (1.7–2.4) ∗∗∗	1.7 (1.4–2.0) ∗∗∗	1.3 (1.1–1.6) ∗	1.2 (1.0–1.5)	1.1 (0.9–1.4)	1.0 (0.8–1.3)	1.6 (1.2–2.1) ∗∗	1.4 (1.1–1.8) ∗	1.4 (1.0–1.9)	1.4 (1.0–1.9)
PTSD (n = 488)	3.8 (2.9–4.8) ∗∗∗	1.9 (1.4–2.5) ∗∗∗	1.8 (1.3–2.5) ∗∗	1.4 (1.0–2.0)	1.3 (0.9–1.8)	0.9 (0.6–1.4)	2.5 (1.7–3.7) ∗∗∗	1.8 (1.2–2.8) ∗∗	1.7 (1.1–2.6) ∗	1.5 (1.0–2.4)
Generalised anxiety disorder (n = 1148)	2.8 (2.3–3.3) ∗∗∗	1.6 (1.3–1.9) ∗∗∗	1.6 (1.2–2.0) ∗∗∗	1.2 (0.9–1.6)	1.5 (1.2–1.9) ∗∗	1.2 (0.9–1.6)	1.4 (1.1–1.9) ∗	1.1 (0.8–1.5)	1.7 (1.2–2.5) ∗∗	1.6 (1.1–2.3) ∗
Major depressive disorder (n = 1716)	2.3 (2.0–2.7) ∗∗∗	1.6 (1.4–1.9) ∗∗∗	1.4 (1.1–1.7) ∗∗	1.2 (0.9–1.5)	1.4 (1.1–1.7) ∗	1.2 (0.9–1.5)	1.4 (1.1–1.8) ∗	1.1 (0.8–1.4)	1.5 (1.1–2.0) ∗	1.4 (1.1–1.9) ∗
Physical health										
Obesity (n = 1794)	1.4 (1.2–1.6) ∗∗∗	1.2 (1.1–1.4) ∗	0.9 (0.8–1.1)	0.9 (0.7–1.1)	1.4 (1.1–1.7) ∗∗	1.3 (1.1–1.6) ∗	1.3 (1.1–1.7) ∗	1.2 (0.9–1.6)	1.1 (0.8–1.5)	1.0 (0.8–1.4)
Diabetes (n = 583)	1.3 (1.1–1.7) ∗	1.2 (1.0–1.5)	1.2 (0.9–1.7)	1.2 (0.8–1.6)	1.3 (1.0–1.9)	1.3 (0.9–1.8)	1.0 (0.7–1.4)	0.9 (0.6–1.4)	0.8 (0.5–1.3)	0.7 (0.5–1.2)
Stroke (n = 131)	1.3 (0.8–2.0)	1.2 (0.8–1.9)	1.6 (0.8–3.1)	1.6 (0.8–3.2)	0.4 (0.2–0.8) ∗	0.4 (0.2–0.8) ∗	1.7 (0.9–3.0)	1.7 (0.9–3.2)	2.1 (1.1–4.3) ∗	2.0 (1.0–4.1)
Heart disease (n = 412)	1.2 (1.0–1.6)	1.3 (1.0–1.7)	0.8 (0.5–1.1)	0.8 (0.5–1.2)	1.4 (0.9–2.1)	1.4 (0.9–2.1)	1.3 (0.8–2.1)	1.3 (0.8–2.2)	0.9 (0.5–1.7)	0.9 (0.5–1.7)
STI (n = 588)	2.1 (1.7–2.6) ∗∗∗	1.5 (1.2–2.0) ∗∗	1.1 (0.9–1.5)	1.0 (0.8–1.4)	1.3 (0.9–1.8)	1.1 (0.8–1.6)	1.5 (1.0–2.2)	1.3 (0.9–1.9)	1.6 (1.1–2.4) ∗	1.5 (1.1–2.3) ∗

Level of significance: ∗∗∗p < 0.001, ∗∗p < 0.01, ∗p < 0.05.

a Model 2 included four CSA subtypes (non-contact, touching, attempted forced intercourse, and completed forced intercourse), each compared with participants who had not experienced that specific subtype, including those with no CSA exposure.

b Model adjusted for age group and gender only.

c Model adjusted for age group, gender, experience of financial hardship during childhood, current financial strain and other types of child maltreatment (physical abuse, emotional abuse, neglect, and exposure to domestic violence) and socio-economic status (based on postcode of residence and quintiles of the Index of Relative Socio-Economic Disadvantage).

### Severity: completed and attempted forced intercourse

Completed forced intercourse was associated with significantly higher odds of health problems compared to those who experienced other forms of CSA (Table 3). This within-CSA comparison helps to disentangle the incremental effects of severity independent of CSA exposure itself. Fully adjusted models showed completed forced intercourse increased odds of outcomes including NSSI (OR 1.8, 95% CI: 1.3–2.6), suicide attempt (OR 1.7, 1.2–2.4), cannabis dependence (OR 2.3, 1.2–4.4), MDD (OR 1.5, 1.1–2.0), and GAD (OR 1.8, 1.2–2.5).

**Table 3:** Association between completed forced intercourse, and attempted and/or completed forced intercourse, and health problems (n = 2348).

Outcomes	Amongst those with CSA			
	Completed forced intercourse vs all other CSA types		Attempted and/or completed forced intercourse vs all other CSA types	
	Simple adjustment odds ratio (95% CI)^a^	Fully adjusted odds ratio (95% CI)^b^	Simple adjustment odds ratio (95% CI)^a^	Fully adjusted odds ratio (95% CI)^b^
Health risk behaviours				
Smoking (n = 1312)	1.4 (1.0–1.9)	1.3 (0.9–1.8)	1.6 (1.1–2.2) ∗∗	1.5 (1.1–2.1) ∗
Binge drinking (n = 868)	1.2 (0.8–1.7)	1.1 (0.7–1.7)	1.3 (0.9–2.0)	1.3 (0.8–1.9)
Cannabis dependence (n = 259)	2.2 (1.2–4.1) ∗	2.3 (1.2–4.4) ∗	1.3 (0.7–2.6)	1.1 (0.5–2.3)
Lifetime NSSI (n = 1676)	2.0 (1.4–2.8) ∗∗∗	1.8 (1.3–2.6) ∗∗∗	1.3 (0.9–1.7)	1.1 (0.8–1.6)
Lifetime suicide attempt (n = 948)	1.9 (1.4–2.7) ∗∗∗	1.7 (1.2–2.4) ∗∗	1.6 (1.1–2.2) ∗	1.3 (0.9–1.9)
Mental disorders				
Any mental disorder (n = 3606)	1.9 (1.4–2.6) ∗∗∗	1.8 (1.3–2.5) ∗∗∗	1.4 (1.1–1.9) ∗	1.3 (1.0–1.7)
PTSD (n = 488)	1.8 (1.2–2.8) ∗∗	1.6 (1.1–2.5) ∗	2.1 (1.3–3.4) ∗∗	1.7 (1.1–2.9) ∗
Generalised anxiety disorder (n = 1148)	1.9 (1.4–2.7) ∗∗∗	1.8 (1.2–2.5) ∗∗	1.2 (0.9–1.8)	1.1 (0.7–1.5)
Major depressive disorder (n = 1716)	1.5 (1.1–2.0) ∗	1.5 (1.1–2.0) ∗	1.1 (0.8–1.5)	1.0 (0.8–1.4)
Alcohol use disorder (n = 1888)	1.2 (0.9–1.7)	1.3 (0.9–1.8)	1.3 (1.0–1.8)	1.3 (0.9–1.7)
Physical health conditions				
Obesity	1.1 (0.8–1.6)	1.0 (0.7–1.4)	1.3 (1.0–1.7)	1.2 (0.9–1.7)
Diabetes (n = 583)	0.9 (0.5–1.5)	0.8 (0.5–1.4)	0.9 (0.6–1.4)	0.9 (0.6–1.4)
Stroke (n = 131)	2.0 (0.8–4.7)	1.9 (0.8–4.3)	1.6 (0.6–4.2)	1.7 (0.6–4.9)
Heart disease (n = 412)	0.8 (0.5–1.5)	0.8 (0.4–1.4)	1.3 (0.8–2.3)	1.4 (0.8–2.4)
STI	1.7 (1.1–2.6) ∗	1.6 (1.1–2.6) ∗	1.3 (0.8–2.0)	1.1 (0.7–1.8)

Level of significance: ∗∗∗p < 0.001, ∗∗p < 0.01, ∗p < 0.05.

a Model adjusted for age group and gender only.

b Model adjusted for age group, gender, experience of financial hardship during childhood, current financial strain, and other types of child maltreatment (physical abuse, emotional abuse, neglect, and exposure to domestic violence).

### Number of CSA perpetrator classes

Results indicate increasing risk associated with multiple perpetrator classes, comparing individuals with CSA by one perpetrator class, and those experiencing CSA by two or more perpetrator classes (Table 4). For example, NSSI prevalence was 9.6% in the no-CSA group, 24.3% in the one-perpetrator group, and 42.0% among those with two or more perpetrator classes. Other significant increases were evident for suicide attempt, any mental disorder, PTSD, GAD, and STI. Supplementary File, Tables S3–4 and S11 provide full details including E-value estimates.

**Table 4:** Prevalence of adult health problems by CSA perpetrator class (any perpetrator; by one perpetrator class only; by two or more perpetrator classes) (n = 8503).

	No CSA (n = 6155)		CSA by any perpetrator (n = 2348)		CSA by one perpetrator class only (n = 1640)		CSA by two or more perpetrator classes (n = 641)	
	n	% (95% CI)	n	% (95% CI)	n	% (95% CI)	n	% (95% CI)
Health risk behaviours								
Smoking (n = 1312)	798	14.8 (13.6–16.0)	514	23.5 (21.3–25.7)	336	22.4 (19.8–25.0)	164	26.6 (22.1–31.1)
Binge drinking (n = 868)	590	10.2 (9.3–11.2)	278	13.0 (11.3–14.8)	192	12.8 (10.7–14.9)	79	13.7 (10.2–17.2)
Cannabis dependence (Scale≥3, n = 259)	129	1.7 (1.3–2.1)	130	4.6 (3.5–5.6)	74	3.8 (2.7–4.9)	52	6.4 (4.1–8.7)
NSSI (n = 1676)	803	9.6 (8.7–10.5)	873	29.4 (27.1–31.6)	529	24.3 (21.8–26.8)	315	42.0 (37.1–46.9)
Suicide attempt (n = 948)	401	5.9 (5.2–6.7)	547	21.8 (19.7–23.9)	309	17.2 (15.0–19.5)	216	33.4 (28.6–38.1)
Mental disorders								
Any mental disorder (n = 3606)	2221	31.8 (30.3–33.3)	1385	53.6 (51.0–56.1)	909	49.5 (46.5–52.6)	436	64.4 (59.6–69.2)
Alcohol use disorder (n = 1888)	1210	17.1 (15.9–18.3)	678	25.3 (23.1–27.5)	462	24.2 (21.7–26.8)	200	28.5 (24.0–33.0)
PTSD (Current) (n = 488)	183	3.1 (2.6–3.7)	305	10.8 (9.3–12.4)	159	8.1 (6.5–9.7)	141	18.7 (15.1–22.4)
Generalised anxiety disorder (n = 1148)	596	8.3 (7.4–9.2)	552	20.1 (18.1–22.1)	333	16.6 (14.4–18.8)	203	29.0 (24.6–33.5)
Major depressive disorder (n = 1716)	1010	14.3 (13.3–15.4)	706	28.5 (26.2–30.8)	465	26.4 (23.8–29.1)	217	33.5 (28.8–38.2)
Physical health conditions								
Obesity (n = 1786)	1189	75.1 (73.6–76.5)	605	31.4 (28.9–33.8)	425	31.3 (28.4–34.2)	162	32.3 (27.5–37.2)
Diabetes (n = 583)	397	9.1 (8.1–10.0)	186	11.6 (9.8–13.4)	140	12.5 (10.3–14.7)	43	10.0 (6.8–13.2)
Stroke (n = 131)	90	2.3 (1.8–2.8)	41	2.7 (1.8–3.6)	29	2.9 (1.8–4.0)	11	2.1 (0.7–3.4)
Heart disease (n = 412)	287	7.0 (6.1–7.9)	125	8.0 (6.5–9.6)	99	8.7 (6.9–10.6)	24	6.5 (3.6–9.3)
STI (n = 586)	314	5.5 (4.8–6.3)	274	11.0 (9.4–12.6)	177	9.9 (8.1–11.7)	94	15.0 (11.5–18.5)

Sixty-seven participants with CSA did not report perpetrator details.

Fig. 1 presents associations between CSA and adult health outcomes (Model 3). Compared to those with no CSA, individuals who experienced CSA had significantly higher odds of adverse outcomes. Those experiencing CSA by two or more perpetrator classes had the worst outcomes, including elevated risk of NSSI (OR 4.1, 3.0–5.5), suicide attempt (OR 3.8 (2.8–5.1), PTSD (OR 2.9, 2.0–4.3), cannabis dependence (OR 2.7, 1.6–4.8), and GAD (OR 2.1, 1.5–2.8). Associations with physical conditions were not significant. Supplementary File, Table S5 provides full details.

### CSA by adult and adolescent perpetrators

Table 5 presents fully adjusted logistic regression models examining associations between CSA involving adult and adolescent perpetrators and adult health outcomes. Those who experienced CSA by both adolescents and adults had higher odds of a range of health problems. Here, the strongest associations were for NSSI: aOR 4.4 (3.0–6.4), suicide attempt (3.4, 2.4–4.9), PTSD (3.2, 2.1–4.9) and STI (2.4, 1.6–3.7) (Supplementary File, Table S6 and S8). Comparison of outcomes between individuals abused by adolescents only, and those abused by adults only, revealed multiple health outcomes for each group were worse compared to those not experiencing CSA, with broadly equivalent risk and effect sizes. Some differences between groups were evident, most notably with those experiencing CSA by adults only at higher risk of suicide attempt, and those experiencing CSA by adolescents only at higher risk of MDD.

**Table 5:** Logistic regression models for CSA by adult and adolescent perpetrator classes (n = 8503).

Outcomes	Simple adjustment odds ratio (95% CI)^a^			Fully adjusted odds ratio (95% CI)^b^			
	CSA by Adolescents only (n = 924) vs No CSA	CSA by Adults only (n = 969) vs No CSA	CSA by both Adolescents and Adults (n = 388) vs No CSA	CSA by Adolescents Only (n = 924) vs No CSA	CSA by Adults Only (n = 969) vs No CSA	CSA by both Adolescents and Adults (n = 388) vs No CSA	CSA by adolescent only (n = 924) vs CSA by adult only (n = 969)
Health risk behaviours							
Binge drinking (n = 865)	1.5 (1.1–2.0) ∗	1.7 (1.3–2.2) ∗∗∗	2.1 (1.4–3.2) ∗∗∗	1.3 (1.0–1.8)	1.5 (1.2–2.0) ∗∗	1.6 (1.1–2.6) ∗	0.9 (0.6–1.3)
Cannabis dependence (n = 256)	3.3 (2.0–5.3) ∗∗∗	3.0 (1.9–4.9) ∗∗∗	4.9 (2.8–8.8) ∗∗∗	2.3 (1.4–3.9) ∗∗	1.9 (1.1–3.2) ∗	2.2 (1.2–4.2) ∗	1.2 (0.7–2.2)
Smoking (n = 1301)	1.7 (1.3–2.1) ∗∗∗	2.0 (1.6–2.5) ∗∗∗	2.5 (1.8–3.5) ∗∗∗	1.4 (1.1–1.8) ∗∗	1.6 (1.3–2.0) ∗∗∗	1.6 (1.1–2.4) ∗	0.9 (0.7–1.2)
NSSI (n = 1658)	3.4 (2.8–4.2) ∗∗∗	3.7 (2.9–4.6) ∗∗∗	8.1 (5.8–11.3) ∗∗∗	2.5 (1.9–3.1) ∗∗∗	2.4 (1.9–3.0) ∗∗∗	4.4 (3.0–6.4) ∗∗∗	1.0 (0.8–1.4)
Suicide attempt (n = 936)	2.8 (2.2–3.7) ∗∗∗	4.4 (3.5–5.6) ∗∗∗	7.1 (5.1–9.9) ∗∗∗	1.9 (1.5–2.6) ∗∗∗	2.8 (2.1–3.6) ∗∗∗	3.4 (2.4–4.9) ∗∗∗	0.7 (0.5–0.9) ∗^,^^c^
Physical health conditions							
Diabetes (n = 580)	1.5 (1.1–2.1) ∗	1.3 (1.0–1.7)	1.3 (0.8–2.1)	1.4 (1.0–2.0)	1.2 (0.9–1.6)	1.1 (0.7–1.8)	1.2 (0.8–1.8)
Heart disease (n = 411)	1.2 (0.8–1.9)	1.3 (1.0–1.8)	1.1 (0.6–2.2)	1.2 (0.8–1.9)	1.3 (1.0–1.9)	1.1 (0.6–2.2)	0.9 (0.6–1.5)
Obesity (n = 1786)	1.1 (0.9–1.3)	1.6 (1.3–1.9) ∗∗∗	1.4 (1.1–1.9) ∗	1.0 (0.8–1.2)	1.4 (1.1–1.6) ∗∗	1.1 (0.8–1.5)	0.7 (0.5–0.9) ∗
Stroke (n = 131)	1.0 (0.5–2.1)	1.5 (0.9–2.5)	0.5 (0.2–1.4)	1.0 (0.5–2.1)	1.4 (0.8–2.3)	0.4 (0.2–1.2)	0.7 (0.3–1.6)
STI (n = 586)	2.2 (1.6–3.0) ∗∗∗	1.8 (1.3–2.4) ∗∗∗	3.7 (2.5–5.4) ∗∗∗	1.8 (1.3–2.4) ∗∗∗	1.3 (0.9–1.8)	2.4 (1.6–3.7) ∗∗∗	1.3 (0.9–1.9)
Mental disorders							
Any mental disorder (n = 3582)	2.8 (2.3–3.4) ∗∗∗	2.2 (1.9–2.6) ∗∗∗	4.9 (3.7–6.5) ∗∗∗	2.1 (1.7–2.6) ∗∗∗	1.5 (1.2–1.8) ∗∗∗	2.7 (2.0–3.7) ∗∗∗	1.4 (1.1–1.8) ∗∗
Alcohol use disorder (n = 1883)	2.2 (1.8–2.8) ∗∗∗	1.8 (1.4–2.1) ∗∗∗	2.8 (2.0–3.7) ∗∗∗	1.9 (1.6–2.4) ∗∗∗	1.4 (1.2–1.8) ∗∗∗	2.1 (1.5–2.9) ∗∗∗	1.3 (1.1–1.8) ∗
Major depressive disorder (n = 1710)	2.6 (2.1–3.2) ∗∗∗	1.9 (1.6–2.3) ∗∗∗	2.8 (2.1–3.7) ∗∗∗	2.0 (1.6–2.4) ∗∗∗	1.3 (1.1–1.6) ∗∗	1.7 (1.2–2.3) ∗∗	1.5 (1.1–1.9) ∗∗
PTSD (n = 483)	2.8 (2.0–4.0) ∗∗∗	3.4 (2.5–4.7) ∗∗∗	8.2 (5.6–11.8) ∗∗∗	1.8 (1.2–2.6) ∗∗	1.8 (1.2–2.5) ∗∗	3.2 (2.1–4.9) ∗∗∗	1.0 (0.7–1.5)
Generalised anxiety disorder (n = 1132)	2.3 (1.8–2.9) ∗∗∗	2.4 (1.9–3.0) ∗∗∗	5.1 (3.7–6.9) ∗∗∗	1.5 (1.2–2.0) ∗∗	1.4 (1.1–1.8) ∗∗	2.3 (1.6–3.4) ∗∗∗	1.1 (0.8–1.5)

Level of significance: ∗∗∗p < 0.001, ∗∗p < 0.01, ∗p < 0.05.

a Model adjusted for age group and gender.

b Model adjusted for age group, gender, financial hardship in childhood, current financial strain, and other CM (CPA, CEA, neglect and EDV).

c For suicide attempt, the odds ratio for adult-only perpetration where the referent group is adolescent-only CSA was 1.4 (1.03–1.9)∗.

### CSA by known and unknown perpetrators

Overall, compared to individuals with no CSA, the highest risks of multiple mental disorders and health risk behaviours (smoking, binge drinking, NSSI, suicide attempt, any mental disorder, GAD, AUD, and PTSD) were present for CSA perpetrated by any known adolescent or any known adult perpetrator (Table 6). Notably, CSA perpetrated by known adults was associated with higher odds of obesity (aOR 1.5), whereas CSA perpetrated by known adolescents was linked to increased odds of STI (aOR 1.8). CSA by unknown adults also presented significant risks. CSA by unknown adolescents presented lower risk, but this group had small cell sizes. Supplementary File, Table S7 and S12 provides full details.

**Table 6:** Fully adjusted odds ratios for health outcomes by Known vs Unknown CSA Perpetrator Type (Adolescent or Adult) (n = 7735).^a^

Outcomes	Adult-perpetrated CSA vs No CSA		Adolescent-perpetrated CSA vs No CSA		Pairwise comparison based on relational perpetrator type		
	Any Known adult (n = 649) vs No CSA OR (95% CI)	Any unknown adult (n = 180) vs No CSA OR (95% CI)	Any Known adolescent (n = 760) vs No CSA OR (95% CI)	Any Unknown adolescent (n = 51) vs No CSA OR (95% CI)	Any known (n = 1409) vs Any unknown (n = 231) OR (95% CI)	Any known adolescent (n = 649) vs Any known adults (n = 760) OR (95% CI)	Any unknown adult (n = 180) vs Any known adult (n = 649) OR (95% CI)
Health risk behaviours							
Binge drinking (n = 782)	1.7 (1.2–2.3) ∗∗	1.1 (0.6–2.0)	1.3 (0.9–1.7)	1.4 (0.5–3.8)	1.2 (0.6–2.2)	0.8 (0.5–1.2)	0.6 (0.3–1.2)
Cannabis dependence (n = 203)	1.8 (0.9–3.3)	1.7 (0.7–4.6)	1.9 (1.1–3.5) ∗	2.7 (0.7–9.6)	0.9 (0.4–2.0)	1.1 (0.5–2.3)	1.0 (0.3–2.8)
Smoking (n = 1134)	1.5 (1.2–2.0) ∗∗	1.7 (1.1–2.6) ∗	1.4 (1.1–1.8) ∗	2.4 (1.1–5.5) ∗	0.7 (0.4–1.2)	0.9 (0.6–1.3)	1.1 (0.7–1.8)
NSSI (n = 1332)	2.3 (1.7–3.1) ∗∗∗	2.3 (1.4–3.9) ∗∗	2.3 (1.8–3.0) ∗∗∗	2.1 (0.8–5.1)	1.1 (0.6–1.8)	1.0 (0.7–1.4)	1.0 (0.6–1.8)
Suicide attempt (n = 710)	2.4 (1.8–3.3) ∗∗∗	3.4 (2.1–5.6) ∗∗∗	1.7 (1.2–2.3) ∗∗	2.5 (1.0–6.5)	0.7 (0.4–1.2)	0.7 (0.5–1.0)	1.4 (0.8–2.4)
Physical health conditions							
Diabetes (n = 573)	1.2 (0.9–1.7)	1.4 (0.7–2.5)	1.3 (0.9–2.0)	4.3 (1.5–11.9) ∗∗	0.5 (0.3–1.0)	1.1 (0.7–1.7)	1.1 (0.6–2.1)
Heart disease (n = 386)	1.4 (1.0–2.1)	1.4 (0.7–3.0)	1.2 (0.7–1.9)	3.1 (0.9–10.5)	0.6 (0.3–1.3)	0.8 (0.5–1.4)	1.0 (0.4–2.2)
Stroke (n = 29)	1.1 (0.6–2.1)	2.3 (0.9–6.0)	1.1 (0.5–2.4)	1.6 (0.2–14.5)	0.6 (0.2–2.0)	1.5 (0.2–14.0)	2.1 (0.7–6.4)
Obese (n = 1614)	1.5 (1.2–1.8) ∗∗∗	1.1 (0.7–1.7)	1.0 (0.8–1.2)	0.9 (0.4–2.2)	1.2 (0.7–1.9)	0.7 (0.5–0.9) ∗∗	0.8 (0.5–1.2)
STI (n = 491)	1.1 (0.7–1.7)	1.6 (0.8–2.9)	1.8 (1.3–2.6) ∗∗∗	1.2 (0.3–4.4)	1.0 (0.5–2.1)	1.6 (1.1–2.5) ∗	1.4 (0.7–2.8)
Mental disorders							
Any mental disorder (n = 3130)	1.3 (1.1–1.7) ∗	1.9 (1.3–2.8) ∗∗	2.1 (1.7–2.6) ∗∗∗	1.2 (0.5–2.9)	1.1 (0.7–1.9)	1.6 (1.2–2.1) ∗∗	1.4 (0.9–2.2)
Alcohol use disorder (n = 1672)	1.4 (1.1–1.9) ∗∗	1.4 (0.9–2.3)	1.9 (1.5–2.4) ∗∗∗	1.6 (0.7–3.3)	1.1 (0.7–1.7)	1.3 (0.9–1.8)	1.0 (0.6–1.6)
Generalised anxiety disorder (n = 929)	1.4 (1.0–1.9) ∗	1.3 (0.8–2.3)	1.4 (1.1–1.9) ∗	1.5 (0.6–3.7)	1.0 (0.6–1.8)	1.0 (0.7–1.4)	0.9 (0.5–1.7)
Major depressive disorder (n = 1475)	1.1 (0.9–1.5)	2.0 (1.3–3.0) ∗∗	1.9 (1.5–2.4) ∗∗∗	1.3 (0.4–3.9)	0.9 (0.5–1.7)	1.7 (1.3–2.3) ∗∗∗	1.8 (1.1–2.8) ∗
PTSD (n = 342)	1.7 (1.1–2.6) ∗	1.4 (0.6–3.3)	1.3 (0.8–2.0)	2.3 (0.8–7.0)	0.8 (0.4–1.7)	0.8 (0.4–1.3)	0.8 (0.4–2.0)

Level of significance: ∗∗∗p < 0.001, ∗∗p < 0.01, ∗p < 0.05.

a Model adjusted for age group, gender, experience of financial hardship during childhood, current financial strain, and other types of CM (CPA, CEA, neglect and EDV). Among the 8503 participants, 67 refused to report the perpetrator type, while 614 participants experienced CSA involving two or more perpetrator classes (these were excluded from the analysis above). CSA by unknown adolescents vs. CSA by known adolescent/unknown adults were not reported in the pairwise comparison due to small sample size (n = 51).

CSA by any known adolescent or any known adult shared equivalent elevated risk for most outcomes, including NSSI (aOR 2.3), and GAD (aOR 1.4). Notably, CSA by any unknown adult presented the highest risk of suicide attempt (aOR 3.4), and high risk of NSSI (aOR 2.3), and MDD (aOR 2.0).

Pairwise comparisons indicated that CSA by known adolescents, compared to known adults, was associated with higher odds of MDD (aOR 1.7) and STI (aOR 1.6).

## Discussion

This study advances the field by employing the most comprehensive approach yet undertaken with a large nationally representative sample to examine associations between CSA severity and perpetrator type, and mental disorders, health risk behaviours and physical conditions in adulthood. Novel insights are further supported by use of robust approaches to measurement of CSA and health outcomes, and by comprehensive adjusting for multiple confounders including other childhood maltreatment.

### Severity

Results confirmed our hypothesis that CSA is strongly associated with health problems in adulthood, with odds of adverse outcomes rising with greater abuse severity. Three findings are notable here. First, comparison between those experiencing CSA with those who do not showed CSA was strongly associated with all four mental disorders (MDD, GAD, PTSD, AUD), all risk behaviours assessed (smoking, binge drinking, cannabis dependence, NSSI, suicide attempt), and two physical conditions (STI, obesity) (Table 1). Moreover, a clear increase in risk emerged when comparing associations by severity via the four types of CSA, confirming findings elsewhere.^8^^,^^37^^,^^38^

Second, more severe types of CSA - e.g., completed and attempted intercourse–had significantly higher odds of multiple health problems (Table 2). Third, comparison between CSA types shows completed intercourse associated with significantly higher odds of several outcomes (cannabis dependence, lifetime NSSI, suicide attempt; PTSD, GAD, MDD) compared to other CSA types, even after adjusting for confounders (Table 3).

The increased risk presented by CSA involving completed sexual penetration, or attempted penetration, and even of sexual contact, is consistent with traumagenic theory.^12^ The elevated risk posed by completed abusive intercourse is particularly notable given its widespread prevalence across the population (8.7%) and the identical prevalence among those aged 16–24 years.^20^ However, this does not diminish the importance of non-penetrative CSA, which is also significantly linked to multiple adverse health outcomes and must not be overlooked in prevention or intervention efforts. As shown here and elsewhere,^31^^,^^34^ any CSA is strongly associated with a myriad of health problems in adulthood. Furthermore, many who experienced completed forced intercourse may have experienced other CSA types, with the more severe type having particular impacts on adult health being significant scientifically, while complete trajectories of risk are relevant for clinical and policy viewpoints.

### Multiple perpetrator types

Results confirmed our hypothesis that CSA by multiple perpetrators is associated with health problems. We found increasing risk for all mental disorders and health risk behaviours, ranging from lowest risk among those with no CSA, to highest risk among those with CSA by multiple perpetrator classes. Those experiencing CSA by two or more perpetrator types had significantly higher odds of PTSD, GAD, NSSI and suicide attempt (Table 4). In addition, CSA involving both adolescent and adult perpetrators was associated with higher odds of mental disorders and risk behaviours, compared to CSA by either adults only, or adolescents only.

These results on the number and constellation of perpetrator types are consistent with traumagenic dynamic theory.^12^ Mental disorders are natural responses to the trauma intrinsic to most instances of CSA, and health risk behaviours are coping mechanisms to deal with these transgressions. Our findings of higher risk comparing one to more than one perpetrator type are important. For those with multiple perpetrator types, the significantly elevated prevalence and risk of PTSD, GAD, NSSI and suicide attempt demonstrate the compounding impact of sexual transgressions. Our findings on obesity differ from those made in a prospective US study of a clinical sample of 160 women, in which 42% of those with CSA were obese compared with 28% in the control group, by age 20–27).^39^ However, the current study draws on nationally representative data and adjusted for other maltreatment types, and other potential confounders. Associations may also be attenuated by high Australian population-wide obesity rates (32%),^40^ self-report measurement,^41^ and sample-wide (vs age-stratified) analysis. Nevertheless, obesity remains an important outcome warranting further analysis.

### Adult and adolescent perpetrators

Results partially supported our hypothesis that CSA by adults and adolescents would be associated with similar health risks. Pairwise comparisons indicated similar elevations in risk, with no consistent differences between groups in NSSI, cannabis dependence, AUD, PTSD, and GAD (Table 5, Table S6). This is consistent with the best available evidence to date.^9^ Equivalence of several heightened risks suggests the traumagenic impact of CSA persists regardless of perpetrator age, which has clinical and normative implications. However, some differences emerged, which add to evidence while illuminating pathways for further research. Compared to those who experienced CSA by adolescents, those experiencing CSA by adults had higher odds of suicide attempt, which could reflect heightened impact on attachment and betrayal trauma. In contrast, adolescent-perpetrated CSA presented higher risk of MDD. These differences between groups were comparatively small, and could be explained by age at the time of victimisation, chronicity, and relational bond with the perpetrator. Nevertheless, they indicate areas of worthwhile further investigation to examine differences in outcomes and consider mechanisms for any such differences potentially related to feelings of helplessness, fear of repetition, guilt, and disrupted relational connections.

### Known and unknown perpetrators

Results generally confirmed our hypothesis that health problems would be greater among those experiencing CSA by known perpetrators. The highest risks of health problems were presented by CSA perpetrated by any known adolescent or any known adult perpetrator, with each of these groups presenting similarly elevated risks. However, CSA by unknown adults also presented significant risks, including the highest risk for suicide attempt, and high risk of NSSI. Overall, results indicate CSA by each of these three groups poses substantial risk for various adult health outcomes.

Several mechanisms might explain why these perpetrator types present elevated risk for adverse adult outcomes. Firstly, CSA by known perpetrators may elicit violation of trust, betrayal trauma, and enduring attachment disruption, each of which is associated with adverse mental health and risk behaviours. This is likely heightened for known perpetrators with close relational ties, such as adult family members.^42^ Secondly, survivors abused by someone they know may experience intensified shame, self-attribution of responsibility, and fear of discovery and risk of blame or denial, which can induce risk behaviours as coping mechanisms, and mental disorders as natural responses. Additionally, known perpetrators may have greater capacity to inflict repeated abuse, leading to cumulative trauma.

Thirdly, the elevated odds of suicide attempt and PTSD posed by CSA by known adults indicates the heightened gravity and trauma of these experiences, and their associated rupturing of hope and psychological security. Fourthly, and similarly, the highest risk for suicide attempt and MDD posed by CSA by an unknown adult suggests these experiences erode perceptions of safety and security, connoting the world itself is dangerous.

### STI

CSA by known adolescents was more strongly associated with increased odds of STI in adulthood, consistent with the longitudinal evidence from Guiney et al., 2024, which highlights pervasive, multi-domain adult health problems following CSA, including elevated STI risk.^7^ This finding is explained by the adolescent-perpetrated sexual abuse disrupting normative sexual development, promoting engagement in high-risk behaviours such as unprotected sex and multiple partners.^7^^,^^43^ Empirical studies consistently show that CSA, especially during adolescence, is linked to earlier sexual debut, higher numbers of sexual partners, and higher STI prevalence across the life course.^5^ The traumagenic dynamics model^13^ elucidates this, positing CSA distorts sexual scripts and induces traumatic sexualisation, powerlessness, and stigmatisation, which motivate risky sexual behaviour and reduce capacity to negotiate safer sex. Together, these findings emphasise the critical need for trauma-informed sexual health interventions targeting survivors of adolescent-perpetrated CSA.

### Strengths and limitations

This study has several strengths. The ACMS dataset involved a large nationally representative sample of individuals aged 16 years and over, enabling population-level assessment of outcomes through life. The ACMS used a robust approach to the concept and operationalisation of CSA, assessed mental disorders with a diagnostic scale, and used validated measures of risk behaviours and physical conditions. This analysis built on these strengths, and in assessing the influence of CSA severity and perpetrator on outcomes, controlled for multiple confounders including other maltreatment types. Using different reference groups allowed us to examine both the overall impact of CSA (vs. no CSA) and the relative severity of risk presented across CSA experiences. This approach reduces the potential dilution effect that would occur if the no-CSA group were included in all severity models.

Limitations should be noted. First, the cross-sectional design precludes causal inference. Reverse causation is possible, where current health status may influence willingness or ability to recall or report CSA. Surveys using retrospective self-report to capture maltreatment are more accurate to those using administrative records such as child protection or criminal justice records,^25^^,^^41^ but participants may not recall events from early childhood and accordingly this form of recall bias may influence results.^44^ Underreporting of CSA among those aged 65 years and over is possible; however, this group comprised only n = 1000 of the sample of N = 8503. Other recall bias through health conditions affecting capacity to accurately report experiences may also limit results. For physical conditions, analysis stratified by age may reveal different outcomes since younger participants (16–24 years) were oversampled and had less time for physical conditions to develop. Small cell sizes for some perpetrators (e.g., unknown adolescents; n = 51) limited precision of estimates for these categories. Second, although the response rate was 14.4%, a low response rate does not necessarily imply selection bias. Survey weights were applied to align the sample with Census distributions and improve representativeness, although residual nonresponse bias cannot be excluded. Co-occurrence of multiple adversities is common. We have adjusted for other maltreatment types as potential confounders, but this may not fully capture their interactive or cumulative effects. Additionally, the number of models and outcomes increases the possibility of chance findings, particularly for inconsistent physical health associations, which should be interpreted with caution. We did not account for CSA chronicity or age of onset, which could influence outcomes. Future research should integrate CSA chronicity and age of onset, distinguish independent and co-occurring maltreatment types, and directly compare self-report and objective measures, to help clarify underlying causal pathways and enhance validity.^41^ In addition, future research could explore important questions to consider the protective factors and characteristics of those who experienced CSA in its various forms, but who did not experience the outcomes assessed. Finally, future research could consider associations with other disorders beyond those assessed here.

### Conclusion

This study found that even after adjusting for multiple confounders including other child maltreatment, CSA remained strongly associated with mental disorders and health risk behaviours; risk increased with CSA severity; risk increased with multiple perpetrators; risk was generally equivalent for both adult-perpetrated and adolescent-perpetrated CSA; and both known and unknown perpetrators of CSA presented significant outcomes. Our findings have implications for prevention and policy across health, education, child protection and legal sectors. While these are diverse, some appear fundamental. Given the prevalence of CSA and its associated harms, investment in prevention of CSA is economically and socially justified. Targeted primary prevention should be accelerated, including through comprehensive sexuality education directed towards the enhancement of protective factors and reduction of risk factors for perpetration of sexual violence in adolescence. Those with lived experience of CSA, whether inflicted by adults or adolescents, require trauma- and violence-informed care. Funding for health and education sectors to provide such care is essential, and support services need to be provided as early as possible. In addition, practitioners across sectors require continuing professional education to build awareness of these outcomes and facilitate enhanced care and prevention.

## Contributors

BM, DF, DH, JS, and HE acquired funding for the study. BM conceived the idea for the study, designed the study, and conducted the literature review. DL processed and validated the data. BM had access to and verified all the study data. HD performed the statistical analyses, with supervision by BM and DL. HD wrote the first draft of the manuscript and BM wrote the revised and final versions. TA and JN reviewed, revised, and contributed to the final manuscript. All authors discussed the results and implications of the study. All authors reviewed and approved the final version of the manuscript, and were responsible for the decision to submit the manuscript for publication.

## Data sharing statement

The ACMS dataset is available to others from July 2026 on a publicly accessible data archive (the Australian Data Archive: https://ada.edu.au/). Anyone who wishes to access the data may do so. The dataset will include deidentified individual participant data and a data dictionary. Additional documentation will also be available (study protocol, approach to statistical analysis). To observe Indigenous Research Principles, we will not make available individual participant data on Indigenous status. For further information, the Lead Investigator of the ACMS can be contacted: b.mathews@qut.edu.au.

## Declaration of interests

We declare no competing interests.
