# Supplementary material for: Perpetrator types, severity, and cumulative trauma: a population-based study of childhood sexual abuse characteristics and lifelong health
Source: Lancet Reg Health West Pac. 2026 Jul 13;72:101923. doi: 10.1016/j.lanwpc.2026.101923 (PMC13382344; doi:10.1016/j.lanwpc.2026.101923)
Supplement: Supplementary Figure and Tables [file mmc1.docx]

**Perpetrator types, severity, and cumulative trauma: a population-based study of childhood sexual abuse characteristics and lifelong health**

**Supplementary file**

Contents

[Figure. S1. Sample and procedures flow 2](#_Toc233703730)

[Table S1: Weighted Prevalence of Health Outcomes by Combination of CSA Subtypes (n=8,503) 3](#_Toc233703731)

[Table S2. Weighted Prevalence of adults with health outcomes, by experience of child sexual abuse subtypes, age group and gender 4](#_Toc233703732)

[Table S3: Weighted Prevalence of outcomes by CSA by adult perpetrator class (n=8,503) 10](#_Toc233703733)

[Table S4: Weighted prevalence of outcomes by CSA by siblings and adolescent perpetrator class (n=8,503) 12](#_Toc233703734)

[Table S5: Association between health outcomes and childhood sexual abuse by CSA by single or multiple perpetrators (n=8,503) 14](#_Toc233703735)

[Table S6: Logistic regression models for CSA with pairwise comparisons by adults and/ or adolescent perpetrator classes (n=7,735)ⱡ 15](#_Toc233703736)

[Table S7: Weighted prevalence of health related outcomes by known/ unknown relationship with CSA perpetrators (n=1,640). 16](#_Toc233703737)

[Table S8: Weighted prevalence of various health related outcomes by adults/ adolescent relationship with CSA perpetrators (n=2281). 17](#_Toc233703738)

[Table S9: Weighted prevalence of various health related outcomes by CSA subtypes (Yes/ No) (n=8,503) 18](#_Toc233703739)

[Table S10: Association between different subtypes of childhood sexual abuse and adult health problems (E-value estimates) 20](#_Toc233703740)

[Table S11: Association between completed forced intercourse, and attempted and/or completed forced intercourse, and health problems (E-value estimates) 22](#_Toc233703741)

[Table S12: Fully adjusted odds ratios for health outcomes by Known vs Unknown CSA Perpetrator Type (Adolescent or Adult) (E-value estimates) 23](#_Toc233703742)

Unknown adults (n=180)

ACMS (n=8,530)

Any CSA

(n=2,348)

No CSA

(n=6,155)

CSA by 2+ perpetrator classes n=708)

Non-contact CSA (n=1,1443)

Any knowns (n=231)

Known adolescents (n=760)

CSA by 1 perpetrator class (n=1,640)

By adult perpetrators only (n=969)

Touching (n=1,525)

Attempt intercourse (n=1,201)

Forced intercourse (n=717)

By adolescent perpetrators only (n=924)

By both adolescent and adult (n=388)

Any unknowns (n=1,409)

Known adults (n=649)

Unknown adolescents (n=51)

Missing

n=67

## Figure. S1. Sample and procedures flow

## Table S1: Weighted Prevalence of Health Outcomes by Combination of CSA Subtypes (n=8,503)

|  | **Non -contact vs any contact CSA** | | | | **Voyeurism and/or Touching vs. Attempted and/or Completed Intercourse** | | | | **Other CSA Types Without Completed Intercourse vs. Completed Intercourse** | | | |
| --- | --- | --- | --- | --- | --- | --- | --- | --- | --- | --- | --- | --- |
| **Outcomes** | **Non-contact CSA only (n=388)** | | **Any touching and/or attempted and/or competed intercourse only (n=1960)** | | **Voyeurism and/ or touching (n=1076)** | | **Attempted and/ or competed intercourse (n=1272)** | | **Other CSA Types without Completed Intercourse (n=1631)** | | **Competed intercourse (n=717)** | |
| **Risk behaviour outcomes** | **n** | **% (95% CI)** | **n** | **% (95% CI)** | **n** | **% (95% CI)** | **n** | **% (95% CI)** | **n** | **% (95% CI)** | **n** | **% (95% CI)** |
| Current smoker (Current) (n=1,312) | 66 | 18.7 (13.6-23.9) | 448 | 24.5 (22.0-26.9) | 187 | 18.1 (15.3-21.0) | 327 | 28.4 (25.2-31.7) | 305 | 20.2 (17.7-22.7) | 209 | 31.1 (26.6-35.5) |
| Binge drinking (n=868) | 43 | 13.6 (9.0-18.1) | 235 | 12.9 (11.0-14.8) | 106 | 11.8 (9.3-14.2) | 172 | 14.1 (11.7-16.6) | 180 | 12.2 (10.2-14.3) | 98 | 14.8 (11.3-18.2) |
| Severe Cannabis dependence (n=259) | 16 | 4.2 (1.5-6.9) | 114 | 4.6 (3.5-5.8) | 36 | 3.0 (1.8-4.3) | 94 | 6.0 (4.4-7.6) | 44 | 2.2 (1.3-3.1) | 54 | 6.2 (4.0-8.4) |
| Lifetime NSSI (n=1,676) | 91 | 21.0 (15.7-26.2) | 782 | 31.1 (28.5-33.6) | 295 | 22.2 (19.2-25.2) | 578 | 35.9 (32.6-39.3) | 505 | 24.0 (21.4-26.5) | 368 | 41.8 (37.2-46.4) |
| Lifetime Suicide attempt (n=948) | 51 | 15.1 (10.2-20.0) | 496 | 23.2 (20.8-25.5) | 151 | 14.3 (11.6-17.0) | 396 | 28.7 (25.5-31.9) | 272 | 16.5 (14.2-18.8) | 275 | 34.1 (29.7-38.4) |
| **Mental disorders** | **n** | **% (95% CI)** | **n** | **% (95% CI)** | **n** | **% (95% CI)** | **n** | **% (95% CI)** | **n** | **% (95% CI)** | **n** | **% (95% CI)** |
| Alcohol use disorder (n=1,888) | 107 | 25.1 (19.8-30.3) | 571 | 25.4 (23.0-27.7) | 268 | 21.7 (18.8-24.7) | 410 | 28.6 (25.4-31.7) | 446 | 23.0 (20.6-25.5) | 232 | 30.5 (26.2-34.8) |
| PTSD (n=488) | 25 | 6.1 (3.1-9.1) | 280 | 11.8 (10.1-13.5) | 70 | 5.5 (3.8-7.1) | 235 | 15.8 (13.3-18.2) | 140 | 7.3 (5.8-8.9) | 165 | 18.9 (15.5-22.3) |
| Generalised anxiety disorder (Current) (n=1,148) | 61 | 12.9 (8.9-16.9) | 491 | 21.5 (19.3-23.7) | 195 | 14.7 (12.1-17.2) | 357 | 25.0 (22.0-28.0) | 317 | 15.8 (13.6-17.9) | 235 | 29.9 (25.6-34.1) |
| Major depressive disorder (lifetime) (n=1,716) | 106 | 25.2 (19.8-30.6) | 600 | 29.2 (26.7-31.7) | 281 | 24.6 (21.4-27.7) | 425 | 32.1 (28.9-35.4) | 449 | 25.3 (22.7-27.9) | 257 | 35.9 (31.4-40.4) |
| Any mental disorder (n=3,606) | 203 | 47.1 (40.9-53.3) | 1182 | 54.9 (52.1-57.7) | 532 | 44.4 (40.7-48.0) | 853 | 62.0 (58.5-65.4) | 873 | 47.2 (44.1-50.2) | 512 | 68.2 (63.8-72.7) |
| **Physical conditions** | **n** | **% (95% CI)** | **n** | **% (95% CI)** | **n** | **% (95% CI)** | **n** | **% (95% CI)** | **n** | **% (95% CI)** | **n** | **% (95% CI)** |
| Obesity (n=1,794) | 85 | 24.5 (19.0-29.9) | 520 | 32.7 (30.0-35.4) | 260 | 27.7 (24.3-31.1) | 345 | 34.7 (31.2-38.2) | 401 | 29.3 (26.4-32.2) | 204 | 36.1 (31.4-40.8) |
| Diabetes (n=583) | 32 | 13.5 (8.7-18.2) | 154 | 11.2 (9.3-13.1) | 96 | 12.9 (10.1-15.6) | 90 | 10.5 (8.2-12.8) | 140 | 12.3 (10.1-14.5) | 46 | 9.9 (6.9-13.0) |
| Stroke (n=131) | 8 | 3.8 (0.9-6.8) | 33 | 2.5 (1.6-3.4) | 11 | 1.7 (0.6-2.9) | 30 | 3.6 (2.2-5.0) | 21 | 2.0 (1.0-2.9) | 20 | 4.4 (2.3-6.5) |
| Heart Disease (n=412) | 16 | 5.7 (2.7-8.8) | 109 | 8.5 (6.7-10.2) | 55 | 7.6 (5.4-9.8) | 70 | 8.4 (6.2-10.6) | 55 | 7.6 (5.4-9.8) | 70 | 8.4 (6.2-10.6) |
| STI (n=588) | 26 | 4.7 (2.4-6.9) | 248 | 12.3 (10.5-14.1) | 85 | 8.3 (6.3-10.4) | 189 | 13.5 (11.1-15.9) | 146 | 8.8 (7.1-10.5) | 128 | 16.1 (12.7-19.4) |

## Table S2. Weighted Prevalence of adults with health outcomes, by experience of child sexual abuse subtypes, age group and gender

| **Gender** | **Age Group** | **No CSA**  **% (95%CI)** | **Any CSA**  **% (95%CI)** | **Non-contact CSA**  **% (95%CI)** | **Any touching**  **% (95%CI)** | **Any attempted forced intercourse**  **% (95%CI)** | **Completed forced intercourse**  **% (95%CI)** |
| --- | --- | --- | --- | --- | --- | --- | --- |
|  | **Current smoking** |  |  |  |  |  |  |
| Women | 16 - 24 years | 7.5 (5.7-9.2) | 21.9 (18.3-25.5) | 23.1 (18.1-28.2) | 24.8 (20.1-29.5) | 25.3 (20.6-30.1) | 31.3 (24.6-37.9) |
| Women | 25 - 44 years | 14.2 (10.9-17.4) | 25.6 (20.6-30.7) | 24.1 (18-30.2) | 25.7 (19.7-31.7) | 32.2 (24.5-39.8) | 32.4 (22.9-42) |
| Women | 45 years or more | 10.2 (8-12.5) | 19.0 (15.3-22.8) | 18.6 (13.8-23.3) | 21.1 (16.4-25.8) | 26.2 (20.2-32.1) | 27.2 (19.6-34.8) |
| Women | Total | 11.2 (9.5-12.8) | 21.7 (19-24.4) | 21.1 (17.8-24.5) | 23.2 (19.9-26.5) | 28.1 (24.1-32.2) | 29.7 (24.4-34.9) |
| Men | 16 - 24 years | 16.0 (14-18) | 27.3 (21-33.7) | 29.9 (21-38.7) | 27.3 (18-36.6) | 30.2 (20.7-39.6) | 38.2 (23.1-53.3) |
| Men | 25 - 44 years | 22.2 (18.9-25.5) | 36.4 (28.6-44.2) | 40.2 (30.4-50) | 32.3 (22.9-41.8) | 40.0 (26.6-53.4) | 46.0 (28.8-63.1) |
| Men | 45 years or more | 15.1 (12.8-17.5) | 20.0 (15-24.9) | 20.8 (14.7-27) | 20.1 (14-26.2) | 24.5 (16.3-32.7) | 28.1 (15.9-40.3) |
| Men | Total | 17.8 (16.1-19.5) | 26.7 (22.7-30.7) | 28.3 (23.2-33.4) | 24.9 (20-29.8) | 29.9 (23.5-36.4) | 35.9 (26.6-45.2) |
|  | **Binge Drinking** |  |  |  |  |  |  |
| Women | 16 - 24 years | 4.8 (3.5-6.1) | 7.8 (5.6-10) | 5.7 (3.2-8.2) | 8.4 (5.6-11.3) | 9.0 (6.1-11.9) | 12.1 (7.6-16.7) |
| Women | 25 - 44 years | 4.1 (2.5-5.7) | 11.1 (7.5-14.7) | 13.1 (8.3-18) | 11.5 (7-16) | 7.3 (3.1-11.6) | 9.6 (3.5-15.7) |
| Women | 45 years or more | 5.8 (4.1-7.5) | 8.7 (6.1-11.3) | 9.5 (5.9-13) | 9.3 (6.1-12.5) | 9.2 (5.7-12.8) | 10.2 (5.1-15.3) |
| Women | Total | 5.1 (4-6.2) | 9.4 (7.5-11.3) | 10.4 (7.8-13) | 10.0 (7.6-12.4) | 8.5 (6.2-10.9) | 10.2 (6.8-13.7) |
| Men | 16 - 24 years | 10.5 (8.9-12.1) | 14.2 (9.6-18.7) | 15.5 (9.1-21.9) | 9.3 (4.2-14.3) | 20.3 (12.2-28.5) | 15.3 (5.7-24.8) |
| Men | 25 - 44 years | 15.1 (12.3-17.9) | 22.1 (15.2-28.9) | 23.4 (14.7-32.1) | 16.9 (9.3-24.5) | 28.7 (16.3-41.1) | 32.7 (16.2-49.3) |
| Men | 45 years or more | 15.3 (13-17.7) | 20.1 (15.1-25.1) | 19.9 (13.8-26) | 21.3 (15.1-27.6) | 28.6 (19.9-37.3) | 27.8 (15.4-40.2) |
| Men | Total | 14.5 (13-16.1) | 20.2 (16.5-23.8) | 20.6 (16.1-25.2) | 18.8 (14.3-23.2) | 27.7 (21.3-34) | 28.2 (19.2-37.2) |
|  | **Cannabis dependence** |  |  |  |  |  |  |
| Women | 16 - 24 years | 1.9 (1-2.8) | 8.4 (6-10.8) | 10.5 (6.9-14.2) | 9.0 (5.9-12.1) | 11.1 (7.7-14.5) | 13.6 (8.7-18.6) |
| Women | 25 - 44 years | 1.2 (0-2.3) | 4.1 (1.9-6.3) | 5.4 (2.2-8.5) | 5.5 (2.4-8.5) | 6.1 (2.4-9.8) | 6.6 (1.9-11.4) |
| Women | 45 years or more | 0.4 (0-0.8) | 1.4 (0.3-2.5) | 1.7 (0.1-3.2) | 1.9 (0.3-3.5) | 1.2 (0.1-2.2) | 2.5 (0-5.1) |
| Women | Total | 0.8 (0.4-1.3) | 3.2 (2.2-4.2) | 4.0 (2.6-5.5) | 4.0 (2.6-5.5) | 4.4 (2.9-6) | 5.5 (3.2-7.8) |
| Men | 16 - 24 years | 4.5 (3.4-5.7) | 11.8 (6.5-17) | 16.6 (8.5-24.7) | 15.3 (6.7-24) | 11.7 (5-18.3) | 20.7 (5.8-35.6) |
| Men | 25 - 44 years | 3.0 (1.6-4.4) | 10.5 (5.3-15.7) | 11.6 (4.9-18.2) | 8.6 (3-14.1) | 16.7 (5.4-27.9) | 20.6 (5.9-35.3) |
| Men | 45 years or more | 1.5 (0.7-2.3) | 3.6 (1.3-5.9) | 3.1 (0.4-5.7) | 3.1 (0.4-5.7) | 6.2 (1.5-10.9) | 7.1 (0-14.2) |
| Men | Total | 2.5 (1.8-3.1) | 6.9 (4.6-9.3) | 7.3 (4.4-10.1) | 6.0 (3.5-8.6) | 10.1 (5.5-14.6) | 13.7 (6.7-20.6) |
|  | **NSSI** |  |  |  |  |  |  |
| Women | 16 - 24 years | 25.0 (22.1-27.9) | 66.2 (62-70.3) | 66.8 (61.2-72.5) | 70.0 (65.1-75) | 69.9 (65-74.8) | 77.8 (72.1-83.6) |
| Women | 25 - 44 years | 16.0 (12.7-19.2) | 41.9 (36.4-47.5) | 44.4 (37.4-51.3) | 44.3 (37.5-51) | 45.7 (37.8-53.7) | 50.6 (40.7-60.4) |
| Women | 45 years or more | 3.5 (2.2-4.7) | 15.2 (11.9-18.5) | 18.2 (13.7-22.7) | 18.3 (14-22.7) | 19.4 (14.4-24.4) | 24.8 (17.9-31.7) |
| Women | Total | 10.5 (9.1-11.9) | 30.9 (28.1-33.8) | 33.5 (29.7-37.2) | 33.9 (30.3-37.5) | 36.4 (32.4-40.5) | 41.5 (36.1-46.8) |
| Men | 16 - 24 years | 16.3 (14.2-18.4) | 41.6 (34.6-48.6) | 37.5 (28.5-46.5) | 52.7 (42.9-62.6) | 55.9 (45.6-66.2) | 49.7 (35.2-64.2) |
| Men | 25 - 44 years | 11.8 (9.3-14.3) | 38.8 (30.9-46.7) | 43.8 (34-53.6) | 35.9 (26.4-45.5) | 47.8 (34-61.5) | 55.3 (38.3-72.4) |
| Men | 45 years or more | 4.0 (2.8-5.3) | 11.2 (7.2-15.1) | 14.4 (9-19.8) | 13.3 (7.9-18.7) | 16.1 (9.5-22.7) | 24.9 (12.4-37.4) |
| Men | Total | 8.7 (7.5-9.8) | 24.3 (20.5-28.2) | 26.6 (21.7-31.5) | 24.5 (19.8-29.3) | 30.5 (24.2-36.9) | 39.1 (29.4-48.7) |
|  | **Lifetime Suicide attempt** |  |  |  |  |  |  |
| Women | 16 - 24 years | 8.4 (6.5-10.3) | 31.2 (27.1-35.3) | 33.6 (28-39.2) | 34.8 (29.6-40) | 35.4 (30.1-40.6) | 46.7 (39.5-53.9) |
| Women | 25 - 44 years | 7.4 (5.1-9.8) | 23.5 (18.6-28.4) | 26.2 (20-32.5) | 25.9 (19.9-32) | 28.4 (21-35.7) | 30.3 (21.1-39.4) |
| Women | 45 years or more | 4.1 (2.7-5.6) | 19.4 (15.8-23) | 22.0 (17.3-26.8) | 22.0 (17.4-26.6) | 25.7 (20.1-31.2) | 30.5 (23.3-37.8) |
| Women | Total | 5.8 (4.7-6.9) | 22.3 (19.7-24.9) | 24.9 (21.5-28.4) | 25.0 (21.7-28.3) | 28.1 (24.3-32) | 32.6 (27.6-37.7) |
| Men | 16 - 24 years | 8.5 (7-10) | 24.9 (19.1-30.8) | 24.3 (16.8-31.8) | 31.2 (22.5-39.9) | 36.6 (26.9-46.3) | 40.4 (26.8-54.1) |
| Men | 25 - 44 years | 7.2 (5.2-9.2) | 23.0 (16.2-29.8) | 26.9 (17.9-35.9) | 23.0 (14.8-31.3) | 37.2 (24.2-50.2) | 43.1 (26.4-59.8) |
| Men | 45 years or more | 4.2 (2.9-5.5) | 15.3 (10.5-20.1) | 15.9 (10-21.8) | 17.5 (11.3-23.7) | 21.8 (13.8-29.8) | 31.4 (17.8-45) |
| Men | Total | 5.9 (4.9-6.9) | 19.1 (15.5-22.7) | 20.5 (15.8-25.1) | 20.6 (16-25.2) | 28.3 (22-34.5) | 36.8 (27.4-46.2) |
|  | **Diabetes** |  |  |  |  |  |  |
| Women | 16 - 24 years | 2.6 (1.6-3.5) | 2.0 (0.9-3.2) | 2.0 (0.5-3.5) | 2.1 (0.6-3.6) | 1.7 (0.5-3) | 0.9 (0-2.2) |
| Women | 25 - 44 years | 4.9 (3-6.8) | 7.8 (4.7-10.9) | 8.7 (4.7-12.8) | 5.9 (2.7-9.2) | 7.4 (3.2-11.6) | 6.0 (1.3-10.8) |
| Women | 45 years or more | 13.6 (11.1-16.1) | 14.5 (11.1-18) | 15.5 (11-19.9) | 16.4 (11.9-20.8) | 12.6 (8.3-17) | 14.9 (9-20.9) |
| Women | Total | 9.2 (7.7-10.7) | 10.6 (8.5-12.8) | 11.4 (8.7-14.2) | 10.9 (8.2-13.5) | 9.1 (6.5-11.8) | 9.8 (6.3-13.3) |
| Men | 16 - 24 years | 1.3 (0.6-2) | 1.1 (0-2.4) | 0.5 (0-1.6) | 2.2 (0-4.7) | 1.4 (0-3.3) | 1.2 (0-3.6) |
| Men | 25 - 44 years | 3.2 (1.9-4.6) | 7.7 (3.3-12.1) | 6.7 (2-11.4) | 11.0 (4.2-17.9) | 9.1 (0-18.3) | 10.9 (0-23.1) |
| Men | 45 years or more | 15.5 (13.1-17.9) | 20.0 (14.8-25.3) | 19.0 (12.8-25.2) | 20.2 (13.9-26.5) | 18.3 (10.7-25.8) | 11.6 (2.6-20.7) |
| Men | Total | 9.0 (7.6-10.3) | 13.6 (10.3-16.9) | 13.0 (9.1-17) | 15.5 (11.2-19.8) | 13.5 (8.3-18.7) | 10.2 (3.6-16.7) |
|  | **Heart disease** |  |  |  |  |  |  |
| Women | 16 - 24 years | 0.5 (0.1-1) | 0.9 (0.2-1.7) | 1.2 (0-2.3) | 0.5 (0-1.2) | 1.2 (0.1-2.2) | 1.3 (0-2.9) |
| Women | 25 - 44 years | 1.0 (0.2-1.9) | 2.1 (0.5-3.6) | 2.3 (0.2-4.3) | 2.6 (0.5-4.7) | 2.2 (0-4.5) | 3.3 (0-6.7) |
| Women | 45 years or more | 9.2 (7.1-11.3) | 12.7 (9.3-16.1) | 11.4 (7.5-15.3) | 12.2 (8.2-16.3) | 11.9 (7.4-16.4) | 9.5 (4.4-14.6) |
| Women | Total | 5.3 (4.2-6.5) | 7.5 (5.6-9.4) | 6.8 (4.7-9) | 7.4 (5-9.7) | 6.8 (4.4-9.3) | 6.1 (3.3-9) |
| Men | 16 - 24 years | 0.5 (0.2-0.9) | 1.4 (0-2.8) | 1.0 (0-2.3) | 0.6 (0-1.7) | 1.9 (0-4.6) | 1.2 (0-3.6) |
| Men | 25 - 44 years | 1.3 (0.4-2.1) | 2.6 (0.1-5.2) | 2.2 (0-5.2) | 3.5 (0-7.4) | 7.8 (0.4-15.2) | 2.6 (0-7.6) |
| Men | 45 years or more | 15.9 (13.5-18.2) | 15.3 (10.7-20) | 12.2 (6.9-17.6) | 17.0 (10.9-23.1) | 17.8 (10.4-25.2) | 20.3 (8-32.6) |
| Men | Total | 8.3 (7-9.5) | 9.3 (6.6-12) | 7.7 (4.5-11) | 10.9 (7.1-14.8) | 12.8 (8-17.7) | 11.6 (4.6-18.5) |
|  | **Obese** |  |  |  |  |  |  |
| Women | 16 - 24 years | 12.0 (10-14) | 16.8 (13.6-20) | 17.9 (13.5-22.2) | 16.7 (12.8-20.7) | 17.8 (13.8-21.9) | 20.5 (14.7-26.3) |
| Women | 25 - 44 years | 22.2 (18.5-25.9) | 28.7 (23.4-33.9) | 31.0 (24.3-37.6) | 29.6 (23.2-35.9) | 33.5 (25.7-41.2) | 34.8 (25.1-44.4) |
| Women | 45 years or more | 25.4 (22.3-28.5) | 36.9 (32.5-41.4) | 35.9 (30.3-41.4) | 41.4 (35.8-46.9) | 41.6 (35.3-47.9) | 44.6 (36.6-52.6) |
| Women | Total | 22.5 (20.4-24.6) | 31.5 (28.5-34.6) | 32.0 (28.2-35.8) | 34.1 (30.4-37.9) | 35.1 (30.9-39.3) | 37.7 (32.3-43.1) |
| Men | 16 - 24 years | 11.1 (9.4-12.8) | 14.4 (9.7-19.2) | 15.5 (8.8-22.2) | 13.0 (7-18.9) | 10.5 (5.1-15.9) | 8.7 (2.2-15.3) |
| Men | 25 - 44 years | 24.2 (20.8-27.6) | 26.2 (19.2-33.2) | 27.6 (19-36.2) | 27.4 (18.3-36.6) | 31.6 (18.7-44.5) | 19.8 (5.5-34.1) |
| Men | 45 years or more | 29.7 (26.7-32.6) | 29.3 (23.7-35) | 27.1 (20.3-33.8) | 35.1 (27.8-42.4) | 31.0 (21.8-40.1) | 35.7 (22.1-49.3) |
| Men | Total | 25.0 (23-26.9) | 26.6 (22.7-30.6) | 26.1 (21.3-31) | 30.5 (25.3-35.8) | 28.7 (22.1-35.4) | 26.7 (17.8-35.7) |
|  | **Stroke** |  |  |  |  |  |  |
| Women | 16 - 24 years | 0.1 (0-0.2) | 0.5 (0-1.1) | 0.8 (0-2) | 0.4 (0-1.1) | 0.7 (0-1.7) | 0.7 (0-2) |
| Women | 25 - 44 years | 0.4 (0-0.8) | 0.3 (0-0.8) | np | 0.4 (0-1.2) | 0.6 (0-1.6) | 0.8 (0-2.4) |
| Women | 45 years or more | 3.4 (2.1-4.7) | 3.9 (2-5.7) | 4.3 (1.7-6.9) | 2.9 (1-4.7) | 4.9 (2.2-7.7) | 6.1 (2.2-9.9) |
| Women | Total | 1.9 (1.2-2.7) | 2.2 (1.2-3.2) | 2.3 (0.9-3.7) | 1.7 (0.7-2.7) | 2.7 (1.3-4.2) | 3.4 (1.4-5.5) |
| Men | 16 - 24 years | 0.3 (0-0.7) | 1.1 (0-3.3) | np | np | 2.6 (0-7.5) | np |
| Men | 25 - 44 years | 0.2 (0-0.6) | 2.8 (0-5.8) | 3.3 (0-7.5) | 1.7 (0-4.3) | 3.0 (0-7.4) | 1.4 (0-4.1) |
| Men | 45 years or more | 5.1 (3.5-6.6) | 5.4 (2.3-8.4) | 7.6 (3.4-11.8) | 6.5 (2.3-10.8) | 10.3 (3.8-16.8) | 14.3 (3.6-25) |
| Men | Total | 2.6 (1.8-3.4) | 4.0 (2-6) | 5.4 (2.6-8.2) | 4.3 (1.7-6.9) | 7.2 (3.1-11.3) | 7.8 (2-13.7) |
|  | **STI** |  |  |  |  |  |  |
| Women | 16 - 24 years | 3.8 (2.7-4.9) | 10.1 (7.6-12.5) | 9.7 (6.4-13.1) | 10.5 (7.3-13.7) | 12.6 (9.2-16) | 14.9 (10-19.9) |
| Women | 25 - 44 years | 8.5 (6-11) | 16.0 (11.7-20.2) | 17.1 (11.8-22.4) | 18.4 (13.1-23.7) | 18.4 (11.9-24.8) | 17.5 (10-25.1) |
| Women | 45 years or more | 4.4 (3-5.9) | 8.2 (5.9-10.5) | 8.8 (5.9-11.8) | 8.4 (5.5-11.4) | 11.0 (7.5-14.5) | 13.7 (8.8-18.5) |
| Women | Total | 5.7 (4.5-6.9) | 11.2 (9.2-13.2) | 12.0 (9.5-14.6) | 12.3 (9.8-14.8) | 13.9 (10.9-16.8) | 15.2 (11.5-19) |
| Men | 16 - 24 years | 2.8 (1.9-3.6) | 11.7 (6.6-16.9) | 9.6 (2.8-16.3) | 11.9 (4.1-19.7) | 16.5 (8.7-24.4) | 26.6 (11.8-41.3) |
| Men | 25 - 44 years | 7.5 (5.5-9.5) | 14.1 (8.5-19.8) | 11.4 (5.4-17.4) | 13.2 (6.2-20.2) | 16.2 (5.4-26.9) | 22.2 (7.4-37) |
| Men | 45 years or more | 4.5 (3.2-5.8) | 8.5 (5.4-11.6) | 8.6 (4.8-12.4) | 9.3 (5.3-13.4) | 11.3 (5.6-17) | 15.2 (5.8-24.5) |
| Men | Total | 5.3 (4.3-6.3) | 10.9 (8.2-13.6) | 9.7 (6.6-12.7) | 10.9 (7.5-14.3) | 13.4 (8.7-18.2) | 19.1 (11.5-26.7) |
|  | **Any mental disorder** |  |  |  |  |  |  |
| Women | 16 - 24 years | 40.0 (36.7-43.2) | 73.8 (70-77.7) | 78.9 (74-83.7) | 73.8 (69-78.7) | 78.0 (73.5-82.5) | 79.0 (73.3-84.7) |
| Women | 25 - 44 years | 35.5 (31.4-39.7) | 62.4 (57-67.8) | 67.0 (60.5-73.5) | 63.6 (57.2-70.1) | 70.4 (63.2-77.6) | 79.2 (71.6-86.9) |
| Women | 45 years or more | 20.3 (17.6-23.1) | 43.6 (39-48.1) | 48.0 (42.1-53.8) | 46.2 (40.6-51.7) | 50.5 (44.1-56.9) | 58.1 (50.1-66.2) |
| Women | Total | 28.0 (25.9-30.1) | 53.9 (50.8-57.1) | 58.6 (54.5-62.6) | 55.8 (51.9-59.6) | 61.7 (57.5-66) | 68.7 (63.6-73.8) |
| Men | 16 - 24 years | 41.2 (38.4-43.9) | 58.1 (50.9-65.3) | 56.7 (47.1-66.4) | 58.9 (49.2-68.5) | 65.0 (54.8-75.2) | 70.0 (57.3-82.7) |
| Men | 25 - 44 years | 42.3 (38.5-46.1) | 65.6 (58-73.1) | 62.3 (52.7-71.9) | 65.5 (55.8-75.2) | 77.0 (65.6-88.4) | 84.0 (72.4-95.7) |
| Men | 45 years or more | 27.5 (24.6-30.3) | 41.3 (35.2-47.4) | 40.6 (33.1-48) | 43.3 (35.8-50.9) | 51.3 (41.6-61) | 54.3 (39.9-68.7) |
| Men | Total | 34.9 (32.8-36.9) | 51.8 (47.3-56.3) | 49.5 (44-55.1) | 52.3 (46.6-58) | 60.8 (53.8-67.8) | 67.1 (57.6-76.7) |
|  | **AUD** |  |  |  |  |  |  |
| Women | 16 - 24 years | 16.3 (13.9-18.6) | 33.9 (29.8-38) | 33.1 (27.5-38.6) | 34.8 (29.7-39.9) | 37.8 (32.5-43) | 35.2 (28.4-42) |
| Women | 25 - 44 years | 13.3 (10.4-16.2) | 29.5 (24.3-34.6) | 29.5 (23.1-35.9) | 27.4 (21.3-33.5) | 31.5 (23.9-39.1) | 32.2 (22.9-41.6) |
| Women | 45 years or more | 7.4 (5.7-9.2) | 15.0 (11.9-18.2) | 16.4 (12.2-20.6) | 15.3 (11.4-19.3) | 18.4 (13.6-23.1) | 22.0 (15.3-28.7) |
| Women | Total | 10.6 (9.2-12) | 22.5 (19.9-25) | 23.2 (19.8-26.5) | 22.0 (18.9-25.2) | 26.0 (22.3-29.7) | 27.5 (22.6-32.4) |
| Men | 16 - 24 years | 26.3 (23.9-28.7) | 32.7 (26.1-39.3) | 32.0 (23.1-40.9) | 36.2 (26.7-45.6) | 37.7 (27.9-47.5) | 45.4 (30.7-60.1) |
| Men | 25 - 44 years | 28.8 (25.3-32.3) | 40.8 (32.9-48.7) | 40.3 (30.7-49.9) | 38.5 (28.7-48.3) | 47.8 (34.1-61.6) | 54.1 (37-71.2) |
| Men | 45 years or more | 16.8 (14.4-19.2) | 23.6 (18.5-28.7) | 21.2 (15.2-27.1) | 23.2 (17-29.4) | 26.7 (18.3-35) | 26.3 (14.5-38.1) |
| Men | Total | 22.6 (20.8-24.4) | 30.7 (26.6-34.8) | 28.7 (23.8-33.7) | 29.6 (24.5-34.6) | 34.5 (27.8-41.2) | 38.8 (29.2-48.4) |
|  | **GAD** |  |  |  |  |  |  |
| Women | 16 - 24 years | 17.2 (14.7-19.7) | 35.7 (31.4-39.9) | 39.8 (33.9-45.6) | 36.6 (31.4-41.9) | 37.2 (31.9-42.5) | 42.6 (35.5-49.7) |
| Women | 25 - 44 years | 11.4 (8.6-14.3) | 24.5 (19.7-29.4) | 26.3 (20.2-32.4) | 26.8 (20.8-32.7) | 31.5 (24.2-38.9) | 36.9 (27.4-46.5) |
| Women | 45 years or more | 4.4 (3-5.8) | 14.4 (11.1-17.6) | 17.1 (12.7-21.6) | 17.1 (12.9-21.3) | 16.6 (11.9-21.3) | 20.6 (14.1-27.2) |
| Women | Total | 8.5 (7.2-9.7) | 20.6 (18.1-23.1) | 23.1 (19.8-26.4) | 22.9 (19.8-26.1) | 25.0 (21.4-28.6) | 29.6 (24.6-34.6) |
| Men | 16 - 24 years | 10.3 (8.5-12.1) | 22.1 (16.5-27.7) | 21.0 (13.8-28.2) | 23.6 (15.8-31.3) | 24.4 (15.9-32.9) | 27.0 (15-39) |
| Men | 25 - 44 years | 10.7 (8.3-13.1) | 24.7 (17.8-31.5) | 26.5 (17.9-35.1) | 23.8 (15.3-32.3) | 34.8 (21.5-48.1) | 39.8 (23-56.7) |
| Men | 45 years or more | 5.2 (3.7-6.7) | 11.8 (7.8-15.7) | 13.2 (8.2-18.3) | 15.6 (10.1-21.2) | 16.4 (9.3-23.5) | 20.3 (9.8-30.7) |
| Men | Total | 8.0 (6.8-9.1) | 17.5 (14.1-20.8) | 18.5 (14.3-22.7) | 19.1 (14.8-23.5) | 23.0 (17-29) | 28.3 (19.6-37) |
|  | **MDD** |  |  |  |  |  |  |
| Women | 16 - 24 years | 19.9 (17.3-22.6) | 35.4 (31.2-39.6) | 36.6 (30.8-42.5) | 34.8 (29.7-39.9) | 35.5 (30.2-40.7) | 34.7 (27.8-41.5) |
| Women | 25 - 44 years | 20.2 (16.8-23.6) | 34.5 (29.2-39.8) | 35.2 (28.5-41.9) | 35.7 (29.2-42.2) | 37.6 (30-45.3) | 42.3 (32.5-52) |
| Women | 45 years or more | 12.2 (10-14.5) | 26.0 (22-30) | 27.6 (22.4-32.7) | 26.6 (21.7-31.4) | 28.1 (22.5-33.6) | 34.1 (26.6-41.7) |
| Women | Total | 15.9 (14.2-17.6) | 30.2 (27.3-33) | 31.4 (27.7-35.2) | 30.9 (27.4-34.4) | 32.6 (28.6-36.5) | 37.2 (31.9-42.5) |
| Men | 16 - 24 years | 16.3 (14.3-18.4) | 23.9 (18-29.8) | 23.6 (15.8-31.5) | 18.6 (11.5-25.6) | 25.4 (16.5-34.3) | 19.5 (9.7-29.4) |
| Men | 25 - 44 years | 15.4 (12.6-18.2) | 33.5 (26-41) | 32.0 (22.9-41.1) | 37.9 (28.1-47.7) | 43.7 (30-57.4) | 45.0 (27.8-62.2) |
| Men | 45 years or more | 10.4 (8.5-12.3) | 20.1 (15.2-24.9) | 21.0 (15-27.1) | 21.2 (15.1-27.2) | 25.3 (17.2-33.4) | 28.6 (16.5-40.7) |
| Men | Total | 13.1 (11.7-14.5) | 25.3 (21.4-29.1) | 25.0 (20.3-29.7) | 26.7 (21.7-31.7) | 30.9 (24.4-37.5) | 33.7 (24.4-42.9) |
|  | **PTSD** |  |  |  |  |  |  |
| Women | 16 - 24 years | 4.3 (2.9-5.6) | 22.7 (19-26.5) | 29.5 (24-35) | 28.6 (23.6-33.6) | 28.0 (23.1-33) | 36.2 (29.1-43.2) |
| Women | 25 - 44 years | 4.3 (2.4-6.1) | 10.8 (7.4-14.1) | 13.6 (9-18.2) | 11.5 (7.4-15.6) | 14.0 (8.7-19.3) | 17.8 (10.6-25.1) |
| Women | 45 years or more | 1.7 (0.8-2.5) | 8.6 (6-11.2) | 8.5 (5.4-11.6) | 8.6 (5.6-11.7) | 12.2 (8.1-16.4) | 13.4 (8-18.8) |
| Women | Total | 2.9 (2.1-3.7) | 11.1 (9.2-13) | 12.8 (10.3-15.2) | 12.1 (9.8-14.3) | 15.3 (12.4-18.2) | 18.1 (14.2-22.1) |
| Men | 16 - 24 years | 2.8 (1.9-3.6) | 9.8 (6-13.6) | 13.3 (7.5-19.1) | 13.3 (7.4-19.1) | 15.0 (8.2-21.9) | 24.1 (12.7-35.4) |
| Men | 25 - 44 years | 4.1 (2.6-5.7) | 10.9 (6-15.8) | 11.8 (5.4-18.2) | 13.6 (6.8-20.4) | 23.1 (11.8-34.5) | 22.5 (8.9-36.2) |
| Men | 45 years or more | 2.7 (1.6-3.9) | 8.7 (5.2-12.2) | 10.1 (5.6-14.7) | 10.4 (5.6-15.1) | 16.0 (8.8-23.2) | 16.7 (6.8-26.5) |
| Men | Total | 3.2 (2.4-4) | 9.6 (7-12.2) | 11.0 (7.6-14.4) | 11.7 (8.1-15.3) | 18.1 (12.6-23.6) | 19.7 (12.4-27) |

np = not published because of small cell size but included in totals where applicable.

## Table S3: Weighted Prevalence of outcomes by CSA by adult perpetrator class (n=8,503)

| **Outcome** | **Class 1: Parents/ caregivers in the home (n=551)** | | | | **Class 2: Institutional caregivers (n=123)** | | | | **Class 3: Other known adults (n=535)** | | | | **Class 4: Unknown adults (n=397)** | | | |
| --- | --- | --- | --- | --- | --- | --- | --- | --- | --- | --- | --- | --- | --- | --- | --- | --- |
|  | **1 class** | | **2+ classes** | | **1 class** | | **2+ classes** | | **1 class** | | **2+ classes** | | **1 class** | | **2+ classes** | |
|  | **n** | **% (95%CI)** | **n** | **% (95%CI)** | **n** | **% (95%CI)** | **n** | **% (95%CI)** | **n** | **% (95%CI)** | **n** | **% (95%CI)** | **n** | **% (95%CI)** | **n** | **% (95%CI)** |
| **Risk behaviour Outcomes** |  |  |  |  |  |  |  |  |  |  |  |  |  |  |  |  |
| Current smoked | 58 | 19.9 (14.5-25.4) | 23 | 7.9 (3.8-11.9) | 15 | 16.6 (7.7-25.5) | 8 | 15.1 (4.2-26.0) | 60 | 27.9 (21.1-34.8) | 76 | 27.5 (20.9-34.1) | 34 | 25.2 (16.8-33.6) | 56 | 30.9 (22.5-39.2) |
| Binge Drinking | 34 | 12.9 (8.5-17.4) | 30 | 13.2 (7.9-18.5) | 8 | 9.3 (2.1-16.5) | 8 | 19.3 (6.1-32.6) | 37 | 18.5 (12.5-24.4) | 37 | 12.5 (7.7-17.3) | 18 | 11.0 (5.3-16.6) | 28 | 13.4 (7.6-19.2) |
| Cannabis dependence | 15 | 3.4 (1.2-5.5) | 18 | 6.2 (2.7-9.8) | 2 | 0.8 (0.0-2.0) | 0 |  | 9 | 4.4 (1.4-7.4) | 73 | 25.4 (18.5-32.2) | 6 | 3.5 (0.6-6.4) | 21 | 6.6 (3.0-10.2) |
| Lifetime NSSI | 96 | 23.2 (17.9-28.5) | 114 | 45.0 (37.1-52.9) | 14 | 12.9 (5.1-20.6) | 17 | 38.3 (21.4-55.1) | 80 | 25.2 (18.9-31.6) | 130 | 41.1 (33.9-48.4) | 48 | 22.4 (14.9-29.8) | 105 | 40.3 (31.9-48.6) |
| Ever attempted suicide | 64 | 17.2 (12.5-22.0) | 92 | 40.1 (32.3-47.9) | 11 | 10.9 (3.1-18.7) | 17 | 34.3 (18.0-50.6) | 60 | 23.1 (16.9-29.4) | 95 | 34.6 (27.6-41.7) | 39 | 21.2 (13.7-28.8) | 73 | 30.4 (22.6-38.2) |
| **Physical conditions** |  |  |  |  |  |  |  |  |  |  |  |  |  |  |  |  |
| Stroke | 3 | 1.7 (0.0-3.8) | 6 | 3.3 (0.5-6.0) | 2 | 2.8 (0.0-7.0) | np |  | 8 | 4.5 (1.1-7.9) | 7 | 2.6 (0.5-4.7) | 6 | 5.3 (1.0-9.6) | 8 | 4.0 (0.9-7.1) |
| Heart disease | 24 | 10.3 (6.0-14.6) | 9 | 4.6 (1.4-7.8) | 8 | 14.3 (4.8-23.9) | 6 | 18.8 (3.9-33.6) | 16 | 9.0 (4.4-13.5) | 14 | 7.3 (3.2-11.5) | 13 | 8.8 (3.1-14.5) | 6 | 4.5 (0.6-8.4) |
| Obese | 100 | 39.8 (33.0-46.5) | 65 | 34.2 (26.4-42.0) | 21 | 28.9 (17.1-40.8) | 11 | 31.5 (15.2-47.8) | 85 | 36.2 (29.0-43.4) | 11 | 31.5 (15.2-47.8) | 45 | 29.2 (21.0-37.4) | 48 | 29.0 (20.8-37.1) |
| Diabetes | 36 | 14.3 (9.5-19.1) | 21 | 11.7 (6.3-17.1) | 7 | 14.9 (4.5-25.2) | 3 | 6.3 (0.0-13.4) | 25 | 10.8 (6.0-15.5) | 25 | 12.0 (7.0-17.1) | 16 | 12.3 (6.1-18.5) | 17 | 10.7 (5.2-16.2) |
| STI | 24 | 6.0 (3.3-8.8) | 38 | 17.9 (11.8-24.0) | 7 | 7.1 (1.5-12.7) | 5 | 7.6 (0.1-15.0) | 24 | 11.1 (6.1-16.1) | 43 | 14.3 (9.4-19.2) | 21 | 10.1 (4.8-15.4) | 42 | 20.0 (13.1-26.8) |
| **Mental disorders** |  |  |  |  |  |  |  |  |  |  |  |  |  |  |  |  |
| PTSD | 38 | 10.0 (6.4-13.7) | 60 | 21.7 (15.5-27.8) | 7 | 7.0 (0.6-13.4) | 9 | 14.4 (4.3-24.5) | 28 | 9.5 (5.4-13.7) | 52 | 16.0 (10.9-21.1) | 14 | 6.6 (2.2-11.0) | 45 | 15.6 (9.9-21.4) |
| GAD | 70 | 16.5 (12.1-20.9) | 78 | 32.7 (25.2-40.2) | 13 | 14.9 (6.1-23.7) | 10 | 13.5 (3.9-23.1) | 47 | 17.2 (11.5-22.9) | 78 | 25.4 (19.2-31.6) | 30 | 14.2 (8.2-20.1) | 81 | 34.0 (25.8-42.2) |
| AUD | 73 | 22.1 (16.8-27.4) | 76 | 30.3 (23.1-37.5) | 15 | 15.6 (6.6-24.7) | 11 | 19.9 (6.8-32.9) | 65 | 22.5 (16.3-28.7) | 77 | 24.2 (18.1-30.3) | 44 | 23.5 (15.9-31.1) | 68 | 29.3 (21.3-37.3) |
| MDD | 75 | 20.6 (15.6-25.7) | 79 | 34.9 (27.3-42.6) | 15 | 17.7 (8.6-26.8) | 14 | 26.8 (12.7-40.9) | 68 | 24.2 (17.9-30.6) | 87 | 30.6 (23.8-37.3) | 53 | 29.4 (21.2-37.6) | 68 | 28.1 (20.7-35.5) |
| Any Mental Disorder | 163 | 44.4 (38.0-50.9) | 166 | 69.4 (62.0-76.7) | 32 | 35.3 (23.3-47.4) | 9 | 18.7 (5.1-32.2) | 133 | 47.6 (40.2-55.0) | 179 | 60.1 (52.8-67.4) | 90 | 51.4 (42.4-60.3) | 152 | 64.9 (56.6-73.2) |

## Table S4: Weighted prevalence of outcomes by CSA by siblings and adolescent perpetrator class (n=8,503)

| **Outcome** | **Class 5: Siblings (n=115)** | | | | **Class 6: Adolescents (current or former romantic partner) (n=966)** | | | | **Class 7: Other known Adolescents (non- romantic) (n=303)** | | | | **Class 8: Unknown Adolescents (n=146)** | | | |
| --- | --- | --- | --- | --- | --- | --- | --- | --- | --- | --- | --- | --- | --- | --- | --- | --- |
|  | **1 class** | | **2+ classes** | | **1 class** | | **2+ classes** | | **1 class** | | **2+ classes** | | **1 class** | | **2+ classes** | |
|  | **n** | **% (95%CI)** | **n** | **% (95%CI)** | **n** | **% (95%CI)** | **n** | **% (95%CI)** | **n** | **% (95%CI)** | **n** | **% (95%CI)** | **n** | **% (95%CI)** | **n** | **% (95%CI)** |
| **Risk behaviour Outcomes** |  |  |  |  |  |  |  |  |  |  |  |  |  |  |  |  |
| Current smoked | 15 | 28.2 (14.7-41.8) | 23 | 45.1 (29.9-60.3) | 24 | 14.6 (7.0-22.1) | 47 | 29.0 (19.6-38.4) | 118 | 20.7 (16.4-25.1) | 89 | 23.3 (17.6-29.1) | 12 | 27.4 (11.6-43.2) | 24 | 27.9 (15.6-40.1) |
| Binge Drinking | 10 | 18.9 (7.2-30.6) | 8 | 17.3 (4.8-29.8) | 14 | 7.7 (2.9-12.5) | 16 | 10.0 (3.2-16.7) | 66 | 10.6 (7.3-13.9) | 48 | 14.7 (9.7-19.6) | 5 | 11.0 (0.9-21.1) | 12 | 16.8 (5.4-28.2) |
| Cannabis dependence | 4 | 7.4 (0.0-14.9) | 5 | 7.4 (0.0-17.0) | 5 | 3.6 (0.0-8.1) | 20 | 10.6 (4.4-16.7) | 29 | 3.8 (1.7-5.9) | 33 | 6.9 (3.9-9.9) | 4 | 4.5 (0.0-9.7) | 13 | 11.4 (4.2-18.6) |
| Lifetime NSSI | 13 | 15.7 (5.8-25.6) | 18 | 23.7 (11.9-35.4) | 52 | 31.1 (21.0-41.2) | 116 | 51.4 (41.5-61.3) | 208 | 27.6 (23.1-32.2) | 202 | 48.4 (41.7-55.1) | 18 | 23.1 (9.3-36.8) | 57 | 56.4 (43.3-69.4) |
| Ever attempted suicide | 10 | 11.4 (3.0-19.9) | 21 | 36.4 (22.2-50.6) | 24 | 14.6 (7.2-22.1) | 77 | 36.0 (27.0-45.0) | 91 | 14.4 (10.8-18.1) | 123 | 34.2 (27.7-40.8) | 10 | 17.6 (4.6-30.5) | 33 | 37.6 (24.9-50.3) |
| **Physical conditions** |  |  |  |  |  |  |  |  |  |  |  |  |  |  |  |  |
| Stroke | 1 | 2.9 (0.0-8.5) | 2 | 3.1 (0.0-7.5) | np |  | 2 | 1.0 (0.0-2.4) | 8 | 2.2 (0.5-4.0) | 2 | 0.5 (0.0-1.2) | 1 | 3.2 (0.0-9.4) | np |  |
| Heart disease | 1 | 1.7 (0.0-5.1) | 4 | 9.6 (0.3-19.0) | 4 | 3.5 (0.0-7.5) | 4 | 4.5 (0.0-9.5) | 27 | 7.5 (4.3-10.7) | 8 | 5.1 (1.1-9.2) | 6 | 15.5 (2.4-28.7) | 2 | 1.6 (0.0-4.0) |
| Obese | 19 | 44.0 (28.1-60.0) | 19 | 31.4 (17.7-45.1) | 16 | 17.2 (7.1-27.3) | 40 | 29.7 (20.2-39.2) | 126 | 24.2 (19.6-28.8) | 87 | 29.6 (23.2-36.0) | 13 | 24.4 (9.0-39.7) | 23 | 33.6 (20.7-46.5) |
| Diabetes | 7 | 22.0 (7.4-36.7) | 6 | 13.2 (2.4-23.9) | 4 | 8.5 (0.0-17.7) | 7 | 5.8 (0.8-10.9) | 4 | 8.5 (0.0-17.7) | 21 | 10.4 (5.9-14.9) | 5 | 27.3 (7.4-47.1) | 4 | 7.0 (0.0-14.0) |
| STI | 4 | 6.2 (0.0-13.4) | 14 | 19.5 (8.7-30.2) | 17 | 18.2 (8.7-27.7) | 26 | 15.6 (8.4-22.8) | 77 | 11.9 (8.4-15.3) | 55 | 14.1 (9.5-18.7) | 3 | 8.2 (0.0-18.1) | 14 | 18.0 (7.1-28.9) |
| **Mental disorders** |  |  |  |  |  |  |  |  |  |  |  |  |  |  |  |  |
| PTSD | 6 | 9.7 (0.0-20.4) | 19 | 29.0 (15.7-42.2) | 15 | 5.6 (2.4-8.8) | 57 | 27.4 (19.1-35.6) | 46 | 6.7 (4.0-9.3) | 84 | 20.4 (15.3-25.5) | 5 | 9.2 (0.6-17.8) | 28 | 27.2 (16.1-38.3) |
| GAD | 9 | 14.2 (2.5-25.9) | 19 | 27.3 (14.3-40.3) | 34 | 19.4 (11.1-27.8) | 70 | 35.3 (26.1-44.5) | 118 | 17.5 (13.5-21.5) | 128 | 33.4 (27.1-39.6) | 12 | 15.4 (4.3-26.6) | 37 | 39.9 (27.1-52.7) |
| AUD | 18 | 32.2 (17.8-46.7) | 18 | 26.4 (12.6-40.1) | 37 | 24.8 (14.9-34.6) | 60 | 32.6 (23.2-42.0) | 194 | 27.8 (23.2-32.4) | 135 | 33.6 (27.2-39.9) | 16 | 22.6 (9.3-35.9) | 34 | 31.2 (19.2-43.2) |
| MDD | 20 | 40.1 (24.4-55.8) | 18 | 31.8 (17.5-46.1) | 46 | 36.0 (24.7-47.2) | 66 | 36.3 (26.8-45.8) | 177 | 29.7 (25.0-34.4) | 135 | 37.4 (30.9-43.9) | 11 | 23.9 (7.3-40.5) | 36 | 38.9 (26.2-51.6) |
| Any Mental Disorder | 36 | 63.7 (48.9-78.5) | 41 | 68.2 (55.0-81.4) | 77 | 56.2 (44.3-68.1) | 138 | 74.7 (66.5-83.0) | 354 | 54.6 (49.3-59.8) | 263 | 68.5 (62.3-74.7) | 14 | 16.1 (5.4-26.7) | 263 | 68.5 (62.3-74.7) |

## Table S5: Association between health outcomes and childhood sexual abuse by CSA by single or multiple perpetrators (n=8,503)

|  | **Any CSA by anyone vs. No CSA** | | **Any CSA by 1 perpetrator class vs. No CSA** | | | **Any CSA by 2+ perpetrator class vs. No CSA** | | |
| --- | --- | --- | --- | --- | --- | --- | --- | --- |
| **Outcomes** | **Simple adjustment odds ratio (95% CI)†** | **Fully adjusted odds ratio (95% CI)‡** | **Simple adjustment odds ratio (95% CI)†** | **Fully adjusted odds ratio (95% CI)‡** | **Simple adjustment odds ratio (95% CI)†** | | **Fully adjusted odds ratio (95% CI)‡** |  |
| **Risk behaviour Outcomes** |  |  |  |  |  | |  |  |
| Current smoker (Current) (n=1,312) | 2.0 (1.7-2.3) *** | 1.6 (1.3-1.9) *** | 1.8 (1.5-2.2) *** | 1.5 (1.2-1.8) *** | 2.4 (1.9-3.1) *** | | 1.6 (1.2-2.2) ** |  |
| Binge drinking (n=868) | 2.3 (1.9-2.7) *** | 1.5 (1.2-1.8) *** | 1.6 (1.3-2.0) *** | 1.4 (1.1-1.8) ** | 2.0 (1.4-2.8) *** | | 1.6 (1.1-2.3) ** |  |
| Severe Cannabis dependence (n=259) | 3.5 (2.5-5.1) *** | 2.4 (1.6-3.6) *** | 2.8 (1.9-4.2) *** | 1.9 (1.2-3.0) ** | 5.4 (3.3-8.9) *** | | 2.7 (1.6-4.8) *** |  |
| Lifetime NSSI (n=1,676) | 4.2 (3.6-5.0) *** | 2.8 (2.4-3.4) *** | 3.2 (2.7-3.9) *** | 2.3 (1.8-2.8) *** | 7.3 (5.6-9.5) *** | | 4.1 (3.0-5.5) *** |  |
| Lifetime Suicide attempt (n=948) | 4.4 (3.6-5.3) *** | 2.7 (2.2-3.4) *** | 3.2 (2.6-3.9) *** | 2.1 (1.7-2.7) *** | 7.4 (5.7-9.7) *** | | 3.8 (2.8-5.1) * |  |
| **Mental disorders** |  |  |  |  |  | |  |  |
| Alcohol use disorder (n=1,888) | 2.0 (1.7-2.4) *** | 1.7 (1.5-2.0) *** | 1.9 (1.6-2.2) *** | 1.6 (1.3-1.9) *** | 2.6 (2.0-3.3) *** | | 2.0 (1.5-2.6) *** |  |
| PTSD (Current) (n=488) | 3.8 (2.9-4.8) *** | 2.1 (1.6-2.7) *** | 2.8 (2.1-3.7) *** | 1.6 (1.2-2.2) ** | 7.1 (5.2-9.8) *** | | 2.9 (2.0-4.3) *** |  |
| GAD (Current) (n=1,148) | 2.8 (2.3-3.3) *** | 1.7 (1.4-2.1) *** | 2.2 (1.8-2.7) *** | 1.4 (1.1-1.8) ** | 4.3 (3.4-5.6) *** | | 2.1 (1.5-2.8) *** |  |
| MDD (lifetime) (n=1,716) | 2.3 (2.0-2.7) *** | 1.6 (1.4-1.9) *** | 2.1 (1.8-2.4) *** | 1.5 (1.3-1.8) *** | 2.8 (2.2-3.5) *** | | 1.8 (1.4-2.3) *** |  |
| Any mental disorder (n=3,606) | 2.7 (2.4-3.1) *** | 1.9 (1.6-2.1) *** | 2.3 (2.0-2.7) *** | 1.6 (1.4-1.9) *** | 4.3 (3.4-5.4) *** | | 2.5 (1.9-3.2) *** |  |
| **Physical conditions** |  |  |  |  |  | |  |  |
| Obesity | 2.1 (1.7-2.6) *** | 1.2 (1.0-1.4) * | 1.3 (1.1-1.6) *** | 1.2 (1.0-1.4) * | 1.4 (1.1-1.8) ** | | 1.1 (0.9-1.5) |  |
| Diabetes (n=583) | 1.3 (1.1-1.7) * | 1.2 (1.0-1.6) | 1.4 (1.1-1.8) ** | 1.3 (1.0-1.7) * | 1.2 (0.8-1.7) | | 1.0 (0.7-1.5) |  |
| Stroke (n=131) | 1.3 (0.8-2.0) | 1.2 (0.8-2.0) | 1.3 (0.8-2.1) | 1.2 (0.7-2.0) | 1.0 (0.5-2.1) | | 0.9 (0.4-1.9) |  |
| Heart Disease (n=412) | 1.2 (1.0-1.6) | 1.3 (1.0-1.7) | 1.3 (1.0-1.7) | 1.3 (1.0-1.8) * | 1.1 (0.6-1.8) | | 1.1 (0.6-1.8) |  |
| STI | 2.1 (1.7-2.6) *** | 1.6 (1.2-2.0) *** | 1.9 (1.5-2.4) *** | 1.5 (1.1-2.0) ** | 3.0 (2.2-4.2) *** | | 2.0 (1.4-2.9) *** |  |

Level of significance: ***p < 0.001, **p < 0.01, *p < 0.05. † Model adjusted for age group and gender only. ‡ Model adjusted for age group, gender, experience of financial hardship during childhood, and other forms of CM (CPA, CEA, neglect and EDV)

## Table S6: Logistic regression models for CSA with pairwise comparisons by adults and/ or adolescent perpetrator classes (n=7,735)ⱡ

| **Outcome** | **Adolescent only vs. Adult only** | | **Both vs. Adolescent only** | | | **Both vs. Adult only** | | |
| --- | --- | --- | --- | --- | --- | --- | --- | --- |
|  | **aOR (95% CI)** | **E-value for point estimate (for CI)** | **aOR (95% CI)** | **E-value for point estimate (for CI)** | **aOR (95% CI)** | | **E-value for point estimate (for CI)** |  |
| **Behavioural domain** |  |  |  |  |  | |  |  |
| Alcohol Binge (n=782) | 0.9 (0.6-1.3) |  | 1.3 (0.8-2.1) |  | 1.1 (0.7-1.7) | |  |  |
| Cannabis dependence (n=203) | 1.2 (0.7-2.2) |  | 1.0 (0.5-1.9) |  | 1.2 (0.6-2.2) | |  |  |
| Current Smoking (n=1,134) | 0.9 (0.7-1.2) |  | 1.2 (0.8-1.7) |  | 1.0 (0.7-1.5) | |  |  |
| Self-Harm Ever (n=1332) | 1.0 (0.8-1.4) |  | 1.8 (1.2-2.7) ** | 3.0 (1.7) | 1.8 (1.2-2.7) ** | | 3.0 (1.7) |  |
| Suicide Attempt Ever (n=710) | 0.7 (0.5-0.9) * or 1.4 (1.03-1.9) * if ref group reversed | 2.2 (1.4) | 1.7 (1.2-2.6) ** | 2.8 (1.7) | 1.2 (0.8-1.8) | |  |  |
| **Physical domain** | 0.9 (0.6-1.5) |  |  |  |  | |  |  |
| Diabetes (n=573) ⱡⱡ | 0.9 (0.6-1.3) |  | 0.8 (0.4-1.4) |  | 0.9 (0.5-1.6) | |  |  |
| Heart Disease (n=386) ⱡⱡ | 1.2 (0.7-2.2) |  | 0.9 (0.4-2.0) |  | 0.8 (0.4-1.7) | |  |  |
| Stroke (n=29) ⱡⱡ | 0.7 (0.3-1.6) |  | 0.4 (0.1-1.5) |  | 0.3 (0.1-0.9) * | | 6.1 (1.5) |  |
| Obese (n=1614) | 0.7 (0.5-0.9) * or 1.4 (1.1-1.8) * reversed | 2.2 (1.4) | 1.1 (0.8-1.6) |  | 0.8 (0.6-1.1) | |  |  |
| STI (n=491) | 1.3 (0.9-1.9) |  | 1.4 (0.9-2.1) |  | 1.8 (1.2-2.8) ** | | 3.0 (1.7) |  |
| **Mental domain** |  |  |  |  |  | |  |  |
| Any mental problem (n=3,130) | 1.4 (1.1-1.8) ** | 1.7 (1.3) | 1.3 (0.9-1.9) |  | 1.9 (1.3-2.6) *** | | 18 (1.3) |  |
| AUD (n=1,672) | 1.3 (1.1-1.8) * | 1.9 (1.4) | 1.1 (0.7-1.5) |  | 1.4 (1.1-2.0) * | | 1.7 (1.3) |  |
| GAD (n=929) | 1.1 (0.8-1.5) |  | 1.5 (1.0-2.3) * |  | 1.7 (1.1-2.5) * | | 2.8 (1.4) |  |
| MDD (n=1,475) | 1.5 (1.1-1.9) ** | 2.4 (1.4) | 0.9 (0.6-1.2) |  | 1.3 (0.9-1.8) | |  |  |
| PTSD (n=342) | 1.0 (0.7-1.5) |  | 1.8 (1.1-2.9) * |  | 1.8 (1.2-2.8) ** | | 3.0 (1.7) |  |

Level of significance: ***p < 0.001, **p < 0.01, *p < 0.05. ‡ Model adjusted for age group, gender, experience of financial hardship during childhood, current financial hardship, and other forms of CM (CPA, CEA, neglect and EDV). ⱡⱡ Omnibus likelihood ratio tests were used to assess the overall effects of CSA predictors. No effects were found for diabetes, heart disease, or stroke (p > 0.05). For outcomes with significant omnibus tests (e.g., PTSD, STI, self-harm, suicide attempt, smoking, binge drinking, MDD, cannabis dependence, AUD), post-hoc LSMEANS with Bonferroni correction confirmed the robustness of the associations. E‑value: minimum strength of association (risk‑ratio scale) that an unmeasured confounder would need to have with both the exposure and the outcome, conditional on measured covariates, to explain away the observed association; E‑value for the CI is the confounder strength required to move the confidence interval to include the null.

## Table S7: Weighted prevalence of health related outcomes by known/ unknown relationship with CSA perpetrators (n=1,640).

|  | **No CSA**  **(n=6,155)** | | | **Any known**  **(n=231)** | | **Any unknown (n=1,409)** | | **Unknown adolescent (n=51)** | | **Known adolescent (n=760)** | | **Known adult**  **(n=649)** | | **Unknown adult (n=180)** | |
| --- | --- | --- | --- | --- | --- | --- | --- | --- | --- | --- | --- | --- | --- | --- | --- |
|  | **n** | **95% CI** | **n** | | **95% CI** | **n** | **95% CI** | **n** | **95% CI** | **n** | **95% CI** | **n** | **95% CI** | **n** | **95% CI** |
| Alcohol Binge | 590 | 10.2 (9.3-11.2) | 169 | | 13.1 (10.8-15.3) | 23 | 11.0 (6.0-15.9) | 5 | 11.0 (0.9-21.1) | 90 | 11.1 (8.2-14.0) | 79 | 14.7 (11.4-18.0) | 18 | 11.0 (5.3-16.6) |
| Cannabis dependence | 129 | 1.7 (1.3-2.1) | 64 | | 3.8 (2.6-5.0) | 10 | 3.7 (1.1-6.3) | 4 | 4.5 (0.0-9.7) | 38 | 4.1 (2.3-6.0) | 26 | 3.5 (1.9-5.1) | 6 | 3.5 (0.6-6.4) |
| Current Smoking | 798 | 14.8 (13.6-16.0) | 290 | | 21.8 (19.1-24.6) | 46 | 25.6 (18.2-33.0) | 12 | 27.4 (11.6-43.2) | 157 | 20.8 (17.0-24.5) | 133 | 22.7 (18.8-26.7) | 34 | 25.2 (16.8-33.6) |
| Diabetes | 397 | 9.1 (8.1-10.0) | 119 | | 12.1 (9.7-14.4) | 21 | 15.1 (8.6-21.7) | 5 | 27.3 (7.4-47.1) | 51 | 11.0 (7.6-14.3) | 68 | 13.0 (9.7-16.2) | 16 | 12.3 (6.1-18.5) |
| Heart Disease | 287 | 7.0 (6.1-7.9) | 80 | | 8.5 (6.5-10.5) | 19 | 10.1 (4.8-15.3) | 6 | 15.5 (2.4-28.7) | 32 | 6.4 (3.8-9.0) | 48 | 10.3 (7.3-13.3) | 13 | 8.8 (3.1-14.5) |
| Obese | 1189 | 24.9 (23.5-26.4) | 367 | | 31.8 (28.6-35.0) | 58 | 28.3 (21.0-35.6) | 13 | 24.4 (9.0-39.7) | 161 | 25.4 (21.2-29.6) | 206 | 37.0 (32.4-41.6) | 45 | 29.2 (21.0-37.4) |
| STI | 314 | 5.5 (4.8-6.3) | 153 | | 9.9 (8.0-11.9) | 24 | 9.7 (5.1-14.4) | 3 | 8.2 (0.0-18.1) | 98 | 12.1 (9.0-15.1) | 55 | 8.2 (5.7-10.7) | 21 | 10.1 (4.8-15.4) |
| Lifetime NSSI | 803 | 9.6 (8.7-10.5) | 463 | | 24.6 (21.9-27.3) | 66 | 22.5 (15.9-29.1) | 18 | 23.1 (9.3-36.8) | 273 | 26.8 (22.9-30.7) | 190 | 22.7 (19.0-26.5) | 48 | 22.4 (14.9-29.8) |
| Stroke | 90 | 2.3 (1.8-2.8) | 22 | | 2.6 (1.4-3.7) | 7 | 4.9 (1.2-8.6) | 1 | 3.2 (0.0-9.4) | 9 | 2.0 (0.6-3.5) | 13 | 3.0 (1.2-4.7) | 6 | 5.3 (1.0-9.6) |
| Suicide Attempt Ever | 119 | 5.9 (5.2-6.7) | 260 | | 16.7 (14.3-19.1) | 49 | 20.5 (13.9-27.1) | 10 | 17.6 (4.6-30.5) | 125 | 14.1 (11.0-17.3) | 135 | 18.8 (15.3-22.3) | 39 | 21.2 (13.7-28.8) |
| Any mental problem | 2221 | 31.8 (30.3-33.3) | 795 | | 49.6 (46.3-52.9) | 114 | 49.2 (41.2-57.2) | 24 | 40.2 (22.4-57.9) | 467 | 55.7 (51.1-60.3) | 328 | 44.6 (40.0-49.2) | 90 | 51.4 (42.4-60.3) |
| AUD | 1210 | 17.1 (15.9-18.3) | 402 | | 24.4 (21.6-27.1) | 60 | 23.3 (16.7-30.0) | 16 | 22.6 (9.3-35.9) | 249 | 27.9 (23.9-31.9) | 153 | 21.5 (17.8-25.2) | 44 | 23.5 (15.9-31.1) |
| GAD | 596 | 8.3 (7.4-9.2) | 291 | | 17.0 (14.6-19.3) | 42 | 14.4 (9.2-19.7) | 12 | 15.4 (4.3-26.5) | 161 | 17.4 (13.9-20.9) | 130 | 16.6 (13.3-19.9) | 30 | 14.2 (8.2-20.1) |
| MDD | 1010 | 14.3 (13.3-15.4) | 401 | | 26.1 (23.3-28.9) | 64 | 28.3 (21.0-35.7) | 11 | 23.9 (7.3-40.5) | 243 | 31.5 (27.3-35.8) | 158 | 21.7 (18.0-25.4) | 53 | 29.4 (21.2-37.6) |
| PTSD | 183 | 3.1 (2.6-3.7) | 140 | | 8.3 (6.5-10.0) | 19 | 7.1 (3.2-11.0) | 5 | 9.2 (0.6-17.7) | 67 | 6.8 (4.5-9.2) | 73 | 9.5 (6.9-12.0) | 14 | 6.6 (2.2-11.0) |

## Table S8: Weighted prevalence of various health related outcomes by adults/ adolescent relationship with CSA perpetrators (n=2281).

| **Outcomes** | **Perpetrators** | **n** | **% (95% CI)** | **Outcomes** | **Perpetrators** | **n** | **% (95% CI)** |
| --- | --- | --- | --- | --- | --- | --- | --- |
| **Current Smoker** | Adolescent only | 192 | 21.2 (17.7-24.7) | **Stroke** | Adolescent only | 10 | 1.9 (0.6-3.2) |
|  | Adult only | 207 | 23.9 (20.6-27.2) |  | Adult only | 25 | 3.6 (2.1-5.2) |
|  | Both adolescent and adult | 101 | 27.2 (21.3-33.1) |  | Both adolescent and adult | 5 | 1.0 (0.1-2.0) |
| **Alcohol Binge** | Adolescent only | 105 | 11.4 (8.7-14.2) | **STI** | Adolescent only | 113 | 11.5 (8.8-14.2) |
|  | Adult only | 117 | 13.9 (11.2-16.5) |  | Adult only | 94 | 9.3 (7.1-11.4) |
|  | Both adolescent and adult | 49 | 13.8 (9.1-18.5) |  | Both adolescent and adult | 64 | 17.5 (12.5-22.4) |
| **Cannabis Dependence** | Adolescent only | 53 | 5.0 (3.1-6.9) | **Any mental problem** | Adolescent only | 575 | 56.4 (52.2-60.6) |
|  | Adult only | 39 | 3.7 (2.3-5.0) |  | Adult only | 507 | 47.4 (43.7-51.2) |
|  | Both adolescent and adult | 34 | 6.1 (3.4-8.7) |  | Both adolescent and adult | 263 | 67.6 (61.6-73.6) |
| **Lifetime NSSI** | Adolescent only | 357 | 28.8 (25.2-32.4) | **AUD** | Adolescent only | 306 | 28.6 (24.9-32.3) |
|  | Adult only | 291 | 24.4 (21.2-27.6) |  | Adult only | 233 | 22.0 (18.9-25.1) |
|  | Both adolescent and adult | 196 | 45.0 (38.5-51.5) |  | Both adolescent and adult | 123 | 29.7 (23.7-35.7) |
| **Suicide Attempt Ever** | Adolescent only | 174 | 16.5 (13.5-19.6) | **GAD** | Adolescent only | 210 | 18.7 (15.5-21.9) |
|  | Adult only | 223 | 21.5 (18.4-24.6) |  | Adult only | 193 | 16.9 (14.2-19.7) |
|  | Both adolescent and adult | 128 | 32.9 (26.6-39.1) |  | Both adolescent and adult | 133 | 32.8 (26.7-38.8) |
| **Diabetes** | Adolescent only | 59 | 11.3 (8.2-14.5) | **MDD** | Adolescent only | 299 | 32.1 (28.2-36.1) |
|  | Adult only | 99 | 12.6 (10.0-15.2) |  | Adult only | 257 | 24.3 (21.2-27.5) |
|  | Both adolescent and adult | 25 | 10.5 (6.2-14.7) |  | Both adolescent and adult | 126 | 33.4 (27.2-39.6) |
| **Heart Disease** | Adolescent only | 40 | 6.6 (4.2-9.0) | **PTSD** | Adolescent only | 101 | 8.7 (6.4-11.1) |
|  | Adult only | 70 | 9.6 (7.2-12.0) |  | Adult only | 106 | 9.4 (7.3-11.5) |
|  | Both adolescent and adult | 13 | 6.5 (2.5-10.5) |  | Both adolescent and adult | 93 | 21.2 (16.2-26.2) |
| **Obese** | Adolescent only | 200 | 25.5 (21.7-29.3) |  |  |  |  |
|  | Adult only | 294 | 35.8 (32.0-39.5) |  |  |  |  |
|  | Both adolescent and adult | 93 | 31.1 (24.8-37.3) |  |  |  |  |

## Table S9: Weighted prevalence of various health related outcomes by CSA subtypes (Yes/ No) (n=8,503)

| Outcomes | CSA subtypes | | | | | | | | |
| --- | --- | --- | --- | --- | --- | --- | --- | --- | --- |
|  | Non-contact CSA | | | Any touching | | Any attempted forced intercourse | | Completed forced intercourse | |
|  |  | n | % (95%CI) | n | % (95%CI) | n | % (95%CI) | n | % (95%CI) |
| Current smoking | No (CSA subtype) | 989 | 15.9 (14.7-17.0) | 967 | 15.8 (14.6-16.9) | 1003 | 15.5 (14.4-16.6) | 1103 | 16.0 (14.9-17.1) |
|  | Yes (CSA subtype) | 323 | 23.9 (21.1-26.7) | 345 | 23.9 (21.2-26.6) | 309 | 28.7 (25.3-32.1) | 209 | 31.1 (26.6-35.5) |
| Alcohol Binge | No (CSA subtype) | 696 | 10.4 (9.4-11.3) | 691 | 10.6 (9.7-11.6) | 708 | 10.6 (9.7-11.6) | 770 | 10.7 (9.8-11.6) |
|  | Yes (CSA subtype) | 172 | 14.1 (11.7-16.4) | 177 | 12.7 (10.6-14.8) | 160 | 13.6 (11.1-16.0) | 98 | 14.8 (11.3-18.2) |
| AUD | No (CSA subtype) | 1474 | 18.1 (17.0-19.2) | 1450 | 18.2 (17.1-19.4) | 1501 | 18.0 (16.9-19.1) | 1656 | 18.4 (17.3-19.5) |
|  | Yes (CSA subtype) | 414 | 25.5 (22.7-28.2) | 438 | 24.5 (21.9-27.2) | 387 | 28.3 (25.1-31.5) | 232 | 30.5 (26.2-34.8) |
| Lifetime NSSI | No (CSA subtype) | 1123 | 11.6 (10.7-12.5) | 1064 | 11.4 (10.5-12.3) | 1129 | 12.0 (11.1-12.9) | 1308 | 12.7 (11.8-13.6) |
|  | Yes (CSA subtype) | 553 | 31.9 (28.9-34.9) | 612 | 31.8 (28.9-34.6) | 547 | 35.7 (32.3-39.1) | 368 | 41.8 (37.2-46.4) |
| Suicide attempt Ever | No (CSA subtype) | 580 | 7.5 (6.7-8.3) | 540 | 7.2 (6.4-8.0) | 572 | 7.5 (6.7-8.3) | 673 | 8.2 (7.4-9.0) |
|  | Yes (CSA subtype) | 368 | 23.9 (21.2-26.7) | 408 | 24.4 (21.7-27.0) | 376 | 28.9 (25.6-32.1) | 275 | 34.1 (29.7-38.4) |
| PTSD | No (CSA subtype) | 262 | 3.7 (3.2-4.3) | 252 | 3.7 (3.1-4.3) | 260 | 3.6 (3.0-4.1) | 323 | 4.0 (3.5-4.6) |
|  | Yes (CSA subtype) | 226 | 12.5 (10.6-14.5) | 236 | 12.3 (10.3-14.2) | 228 | 16.3 (13.7-18.8) | 165 | 18.9 (15.5-22.3) |
| GAD | No (CSA subtype) | 779 | 9.3 (8.5-10.2) | 757 | 9.1 (8.3-10.0) | 806 | 9.5 (8.7-10.4) | 913 | 9.9 (9.1-10.8) |
|  | Yes (CSA subtype) | 369 | 22.3 (19.7-24.9) | 391 | 22.5 (19.9-25.0) | 342 | 25.1 (22.1-28.2) | 235 | 29.9 (25.6-34.1) |
| MDD | No (CSA subtype) | 1277 | 16.0 (14.9-17.1) | 1257 | 15.8 (14.7-16.9) | 1316 | 16.2 (15.2-17.3) | 1459 | 16.7 (15.7-17.7) |
|  | Yes (CSA subtype) | 439 | 29.1 (26.2-32.0) | 459 | 29.4 (26.6-32.3) | 400 | 31.9 (28.6-35.3) | 257 | 35.9 (31.4-40.4) |
| Any mental disorder | No (CSA subtype) | 2725 | 34.0 (32.6-35.4) | 2692 | 34.0 (32.6-35.4) | 2797 | 34.2 (32.8-35.6) | 3094 | 35.1 (33.8-36.5) |
|  | Yes (CSA subtype) | 881 | 56.0 (52.7-59.2) | 914 | 55.1 (52.0-58.2) | 809 | 61.8 (58.2-65.4) | 512 | 68.2 (63.8-72.7) |
| Stroke | No (CSA subtype) | 101 | 2.2 (1.7-2.7) | 107 | 2.4 (1.9-2.9) | 102 | 2.2 (1.7-2.7) | 111 | 2.2 (1.8-2.7) |
|  | Yes (CSA subtype) | 30 | 3.3 (2.0-4.6) | 24 | 2.4 (1.4-3.4) | 29 | 3.8 (2.3-5.3) | 20 | 4.4 (2.3-6.5) |
| Heart Disease | No (CSA subtype) | 339 | 7.3 (6.5-8.2) | 330 | 7.0 (6.2-7.9) | 346 | 7.1 (6.3-8.0) | 376 | 7.3 (6.5-8.1) |
|  | Yes (CSA subtype) | 73 | 7.2 (5.4-9.0) | 82 | 8.4 (6.4-10.4) | 66 | 8.3 (6.1-10.4) | 36 | 7.6 (4.8-10.4) |
| Cannabis Dependence | No (CSA subtype) | 165 | 1.9 (1.5-2.3) | 169 | 2.0 (1.6-2.5) | 168 | 2.0 (1.6-2.4) | 192 | 2.1 (1.7-2.5) |
|  | Yes (CSA subtype) | 94 | 5.4 (4.0-6.8) | 90 | 4.7 (3.4-5.9) | 91 | 6.0 (4.4-7.7) | 67 | 7.6 (5.2-10.0) |
| Obese | No (CSA subtype) | 1409 | 24.7 (23.4-26.0) | 1370 | 25.0 (23.6-26.4) | 1468 | 25.5 (24.1-26.8) | 1590 | 25.9 (24.6-27.2) |
|  | Yes (CSA subtype) | 385 | 30.1 (27.1-33.1) | 424 | 34.3 (31.2-37.4) | 326 | 35.1 (31.4-38.7) | 204 | 36.1 (31.4-40.8) |
| Diabetes | No (CSA subtype) | 459 | 9.3 (8.3-10.2) | 451 | 9.2 (8.3-10.1) | 497 | 9.7 (8.7-10.6) | 537 | 9.8 (8.9-10.7) |
|  | Yes (CSA subtype) | 124 | 12.1 (9.8-14.3) | 132 | 12.3 (10.1-14.5) | 86 | 10.5 (8.1-12.9) | 46 | 9.9 (6.9-13.0) |
| STI | No (CSA subtype) | 414 | 6.2 (5.4-6.9) | 405 | 6.0 (5.3-6.7) | 410 | 6.0 (5.3-6.7) | 460 | 6.2 (5.5-6.9) |
|  | Yes (CSA subtype) | 174 | 11.2 (9.3-13.1) | 183 | 11.8 (9.8-13.8) | 178 | 13.7 (11.2-16.1) | 128 | 16.1 (12.8-19.4) |

## Table S10: Association between different subtypes of childhood sexual abuse and adult health problems (E-value estimates)

| **Outcomes** | **Model 1: Any CSA vs No CSA** | | **Model 2: CSA subtypes vs. no exposure to that subtype including those with no CSA exposure §** | | | | | | | | |
| --- | --- | --- | --- | --- | --- | --- | --- | --- | --- | --- | --- |
|  |  |  | **Non-contact CSA vs. no non-contact CSA** | | | **Touching vs. no touching** | | **Attempted forced intercourse vs. no attempted forced intercourse** | | **Completed forced intercourse vs. no completed forced intercourse** | |
|  | **Fully adjusted odds ratio (95% CI)‡** | **E-value for point estimate (for CI)** | **Fully adjusted odds ratio (95% CI)‡** | **E-value for point estimate (for CI)** | **Fully adjusted odds ratio (95% CI)‡** | | **E-value for point estimate (for CI)** | **Fully adjusted odds ratio (95% CI)‡** | **E-value for point estimate (for CI)** | **Fully adjusted odds ratio (95% CI)‡** | **E-value for point estimate (for CI)** |
| **Health risk behaviours** |  |  |  |  |  | |  |  |  |  |  |
| Current smoker (n=1,312) | 1.5 (1.3-1.8) *** | 1.75 (1.54) | 1.1 (0.9-1.4) |  | 1.0 (0.8-1.3) | |  | 1.5 (1.1-2.1) ** | 1.8 (1.3) | 1.3 (0.9-1.9) |  |
| Binge drinking (n=868) | 1.4 (1.2-1.8) *** | 1.65 (1.8) | 1.4 (1.0-1.8) * | 1.7 (1.3) | 1.0 (0.7-1.3) | |  | 1.1 (0.8-1.7) |  | 1.3 (0.8-2.0) |  |
| Cannabis dependence (n=259) | 2.2 (1.4-3.4) *** | 3.8 (2.2) | 1.9 (1.1-3.1) * | 3.2 (1.4) | 0.8 (0.4-1.5) | |  | 1.2 (0.6-2.5) |  | 2.2 (1.1-4.5) * | 3.8 (1.4) |
| Lifetime non-suicidal self-injury (n=1,676) | 2.8 (2.3-3.3) *** | 5.0 (4.0) | 1.7 (1.3-2.2) *** | 2.8 (1.9) | 1.4 (1.1-1.8) ** | | 2.2 (1.4) | 1.3 (1.0-1.8) |  | 1.7 (1.2-2.5) ** | 2.8 (1.7) |
| Lifetime suicide attempt (n=948) | 2.7 (2.2-3.3) *** | 4.8 (3.8) | 1.5 (1.1-2.0) * | 2.4 (1.4) | 1.3 (1.0-1.8) | |  | 1.5 (1.1-2.1) ** | 2.4 (1.4) | 1.6 (1.1-2.3) ** | 2.6 (1.4) |
| **Mental disorders** |  |  |  |  |  | |  |  |  |  |  |
| Any mental disorder (n=3,606) | 1.8 (1.6-2.1) *** | 2.0 (1.8) | 1.3 (1.1-1.6) ** | 1.5 (1.3) | 1.1 (0.9-1.3) | |  | 1.4 (1.1-1.8) * | 1.7 (1.3) | 1.8 (1.3-2.5) *** | 2.0 (1.5) |
| Alcohol use disorder (n=1,888) | 1.7 (1.4-2.0) *** | 1.9 (1.6) | 1.2 (1.0-1.5) |  | 1.0 (0.8-1.3) | |  | 1.4 (1.0-1.8) * | 1.7 (1.2) | 1.4 (1.0-1.9) |  |
| PTSD (n=488) | 1.9 (1.4-2.5) *** | 3.2 (2.1) | 1.4 (1.0-2.0) |  | 0.9 (0.6-1.4) | |  | 1.8 (1.2-2.8) ** | 3 (1.7) | 1.5 (1.0-2.4) |  |
| Generalised anxiety disorder (n=1,148) | 1.6 (1.3-1.9) *** | 1.8 (1.5) | 1.2 (0.9-1.6) |  | 1.2 (0.9-1.6) | |  | 1.1 (0.8-1.5) |  | 1.6 (1.1-2.3) * | 2.6 (1.4) |
| Major depressive disorder (n=1,716) | 1.6 (1.4-1.9) *** | 1.8 (1.7) | 1.2 (0.9-1.5) |  | 1.2 (0.9-1.5) | |  | 1.1 (0.8-1.4) |  | 1.4 (1.1-1.9) * | 2.2 (1.4) |
| **Physical health** |  |  |  |  |  | |  |  |  |  |  |
| Obesity (n=1,794) | 1.2 (1.0-1.4) * | 1.7 (1.4) | 0.9 (0.7-1.1) |  | 1.3 (1.1-1.6) * | | 1.9 (1.4) | 1.2 (0.9-1.6) |  | 1.0 (0.8-1.4) |  |
| Diabetes (n=583) | 1.2 (1.0-1.5) |  | 1.2 (0.8-1.6) |  | 1.3 (0.9-1.8) | |  | 0.9 (0.6-1.4) |  | 0.7 (0.5-1.2) |  |
| Stroke (n=131) | 1.2 (0.8-1.9) |  | 1.6 (0.8-3.2) |  | 0.4 (0.2-0.8) * | | 4.4 (1.8) | 1.7 (0.9-3.2) |  | 2.0 (1.0-4.1) |  |
| Heart disease (n=412) | 1.3 (1.0-1.7) |  | 0.8 (0.5-1.2) |  | 1.4 (0.9-2.1) | |  | 1.3 (0.8-2.2) |  | 0.9 (0.5-1.7) |  |
| STI (n=588) | 1.5 (1.2-2.0) ** | 2.4 (1.7) | 1.0 (0.8-1.4) |  | 1.1 (0.8-1.6) | |  | 1.3 (0.9-1.9) |  | 1.5 (1.1-2.3) * | 2.4 (1.4) |

§Model 2 included four CSA subtypes (non-contact, touching, attempted forced intercourse, and completed forced intercourse), each compared with participants who had not experienced that specific subtype, including those with no CSA exposure.

† Model adjusted for age group and gender only.

‡ Model adjusted for age group, gender, experience of financial hardship during childhood, current financial strain and other types of child maltreatment (physical abuse, emotional abuse, neglect, and exposure to domestic violence) and socio-economic status (based on postcode of residence and quintiles of the Index of Relative Socio-Economic Disadvantage).

Level of significance: ***p < 0.001, **p < 0.01, *p < 0.05.

E‑value: minimum strength of association (risk‑ratio scale) that an unmeasured confounder would need to have with both the exposure and the outcome, conditional on measured covariates, to explain away the observed association; E‑value for the CI is the confounder strength required to move the confidence interval to include the null. For example, the E‑value for the association between completed forced intercourse and cannabis dependence is 3.8 (E‑value for CI = 2.2), indicating an unmeasured confounder would need risk‑ratio associations of at least 3.8 with both exposure and outcome to fully explain the point estimate, and at least 2.2 to move the CI to include the null.

## Table S11: Association between completed forced intercourse, and attempted and/or completed forced intercourse, and health problems (E-value estimates)

| **Outcomes** | **Amongst those with CSA** | | | |
| --- | --- | --- | --- | --- |
|  | **Completed forced intercourse vs all other CSA types** | | **Completed forced intercourse vs all other CSA types** | |
|  | **Fully adjusted odds ratio (95% CI)‡** | **E-value for point estimate (for CI)** | **Fully adjusted odds ratio (95% CI)‡** | **E-value for point estimate (for CI)** |
| **Health risk behaviours** |  |  |  |  |
| Smoking (n=1,312) | 1.3 (0.9-1.8) |  | 1.5 (1.1-2.1) * | 1.8 (1.3) |
| Binge drinking (n=868) | 1.1 (0.7-1.7) |  | 1.3 (0.8-1.9) |  |
| Cannabis dependence (n=259) | 2.3 (1.2-4.4) * | 4.0 (1.7) | 1.1 (0.5-2.3) |  |
| Lifetime NSSI (n=1,676) | 1.8 (1.3-2.6) *** | 3.0 (1.9) | 1.1 (0.8-1.6) |  |
| Lifetime suicide attempt (n=948) | 1.7 (1.2-2.4) ** | 2.8 (1.7) | 1.3 (0.9-1.9) |  |
| **Mental disorders** |  |  |  |  |
| Any mental disorder (n=3,606) | 1.8 (1.3-2.5) *** | 2.0 (1.5) | 1.3 (1.0-1.7) |  |
| PTSD (n=488) | 1.6 (1.1-2.5) * | 2.6 (1.4) | 1.7 (1.1-2.9) * | 2.8 (1.4) |
| Generalised anxiety disorder (n=1,148) | 1.8 (1.2-2.5) ** | 3.0 (1.7) | 1.1 (0.7-1.5) |  |
| Major depressive disorder (n=1,716) | 1.5 (1.1-2.0) * | 2.4 (1.4) | 1.0 (0.8-1.4) |  |
| Alcohol use disorder (n=1,888) | 1.3 (0.9-1.8) |  | 1.3 (0.9-1.7) |  |
| **Physical health conditions** |  |  |  |  |
| Obesity | 1.0 (0.7-1.4) |  | 1.2 (0.9-1.7) |  |
| Diabetes (n=583) | 0.8 (0.5-1.4) |  | 0.9 (0.6-1.4) |  |
| Stroke (n=131) | 1.9 (0.8-4.3) |  | 1.7 (0.6-4.9) |  |
| Heart disease (n=412) | 0.8 (0.4-1.4) |  | 1.4 (0.8-2.4) |  |
| STI | 1.6 (1.1-2.6) * | 2.6 (1.4) | 1.1 (0.7-1.8) |  |

Level of significance: ***p < 0.001, **p < 0.01, *p < 0.05. † Model adjusted for age group and gender only. ‡ Model adjusted for age group, gender, experience of financial hardship during childhood, current financial strain, and other types of child maltreatment (physical abuse, emotional abuse, neglect, and exposure to domestic violence). E‑value: minimum strength of association (risk‑ratio scale) that an unmeasured confounder would need to have with both the exposure and the outcome, conditional on measured covariates, to explain away the observed association; E‑value for the CI is the confounder strength required to move the confidence interval to include the null.

## Table S12: Fully adjusted odds ratios for health outcomes by Known vs Unknown CSA Perpetrator Type (Adolescent or Adult) (E-value estimates)

|  | **Adult-perpetrated CSA vs No CSA** | | **Adolescent-perpetrated CSA vs No CSA** | | **Pairwise comparison based on relational perpetrator type** | | |
| --- | --- | --- | --- | --- | --- | --- | --- |
| **Outcomes** | **Any Known adult (n=649) vs No CSA**  **OR (95%CI)**  **[E-value]** | **Any unknown adult (n=180) vs No CSA**  **OR (95%CI)** | **Any Known adolescent (n=760) vs No CSA**  **OR (95%CI)** | **Any Unknown adolescent (n=51) vs No CSA**  **OR (95%CI)** | **Any known (n=1,409) vs Any unknown (n=231)**  **OR (95%CI)** | **Any known adolescent (n=649) vs Any known adults (n=760)**  **OR (95%CI)** | **Any unknown adult (n=180) vs Any known adult (n=649)**  **OR (95%CI)** |
| **Health risk behaviours** |  |  |  |  |  |  |  |
| Binge drinking (n=782) | 1.7 (1.2-2.3) **  [1.9 (1.4)] | 1.1 (0.6-2.0) | 1.3 (0.9-1.7) | 1.4 (0.5-3.8) | 1.2 (0.6-2.2) | 0.8 (0.5-1.2) | 0.6 (0.3-1.2) |
| Cannabis dependence (n=203) | 1.8 (0.9-3.3) | 1.7 (0.7-4.6) | 1.9 (1.1-3.5) *  [3.2 (1.4)] | 2.7 (0.7-9.6) | 0.9 (0.4-2.0) | 1.1 (0.5-2.3) | 1.0 (0.3-2.8) |
| Smoking (n=1,134) | 1.5 (1.2-2.0) **  [1.8 (1.4)] | 1.7 (1.1-2.6) *  [1.9 (1.3)] | 1.4 (1.1-1.8) *  [1.7 (1.3)] | 2.4 (1.1-5.5) *  [2.3 (1.3)] | 0.7 (0.4-1.2) | 0.9 (0.6-1.3) | 1.1 (0.7-1.8) |
| Non-suicidal self-injury (n=1,332) | 2.3 (1.7-3.1) ***  [4.0 (2.8)] | 2.3 (1.4-3.9) **  [4.0 (2.1)] | 2.3 (1.8-3.0) ***  [4.0 (3.0)] | 2.1 (0.8-5.1) | 1.1 (0.6-1.8) | 1.0 (0.7-1.4) | 1.0 (0.6-1.8) |
| Suicide attempt (n=710) | 2.4 (1.8-3.3) ***  [4.2 (3.0)] | 3.4 (2.1-5.6) ***  [6.2 (3.6)] | 1.7 (1.2-2.3) **  [2.8 (1.7)] | 2.5 (1.0-6.5) | 0.7 (0.4-1.2) | 0.7 (0.5-1.0) | 1.4 (0.8-2.4) |
| **Physical health conditions** |  |  |  |  |  |  |  |
| Diabetes (n=573) | 1.2 (0.9-1.7) | 1.4 (0.7-2.5) | 1.3 (0.9-2.0) | 4.3 (1.5-11.9) **  [8.1 (2.4)] | 0.5 (0.3-1.0) | 1.1 (0.7-1.7) | 1.1 (0.6-2.1) |
| Heart disease (n=386) | 1.4 (1.0-2.1) | 1.4 (0.7-3.0) | 1.2 (0.7-1.9) | 3.1 (0.9-10.5) | 0.6 (0.3-1.3) | 0.8 (0.5-1.4) | 1.0 (0.4-2.2) |
| Stroke (n=29) | 1.1 (0.6-2.1) | 2.3 (0.9-6.0) | 1.1 (0.5-2.4) | 1.6 (0.2-14.5) | 0.6 (0.2-2.0) | 1.5 (0.2-14.0) | 2.1 (0.7-6.4) |
| Obese (n=1,614) | 1.5 (1.2-1.8) ***  [2.7 (1.7)] | 1.1 (0.7-1.7) | 1.0 (0.8-1.2) | 0.9 (0.4-2.2) | 1.2 (0.7-1.9) | 0.7 (0.5-0.9) **  [2.2 (1.5)] | 0.8 (0.5-1.2) |
| STI (n=491) | 1.1 (0.7-1.7) | 1.6 (0.8-2.9) | 1.8 (1.3-2.6) ***  [3.0 (1.9)] | 1.2 (0.3-4.4) | 1.0 (0.5-2.1) | 1.6 (1.1-2.5) *  [2.6 (1.4)] | 1.4 (0.7-2.8) |
| **Mental disorders** |  |  |  |  |  |  |  |
| Any mental disorder (n=3,130) | 1.3 (1.1-1.7) *  [1.5 (1.3)] | 1.9 (1.3-2.8) **  [2.1 (1.5)] | 2.1 (1.7-2.6) ***  [2.3 (1.9)] | 1.2 (0.5-2.9) | 1.1 (0.7-1.9) | 1.6 (1.2-2.1) **  [1.8 (1.4)] | 1.4 (0.9-2.2) |
| Alcohol use disorder (n=1,672) | 1.4 (1.1-1.9) **  [1.7 (1.3)] | 1.4 (0.9-2.3) | 1.9 (1.5-2.4) ***  [2.1 (1.8)] | 1.6 (0.7-3.3) | 1.1 (0.7-1.7) | 1.3 (0.9-1.8) | 1.0 (0.6-1.6) |
| Generalised anxiety disorder (n=929) | 1.4 (1.1-1.9) *  [2.2 (1.4)] | 1.3 (0.8-2.3) | 1.4 (1.1-1.9) *  [2.2 (1.4)] | 1.5 (0.6-3.7) | 1.0 (0.6-1.8) | 1.0 (0.7-1.4) | 0.9 (0.5-1.7) |
| Major depressive disorder (n=1,475) | 1.1 (0.9-1.5) | 2.0 (1.3-3.0) **  [3.4 (1.9)] | 1.9 (1.5-2.4) ***  [3.2 (2.4)] | 1.3 (0.4-3.9) | 0.9 (0.5-1.7) | 1.7 (1.3-2.3) ***  [2.8 (1.9)] | 1.8 (1.1-2.8) *  [3.0 (1.4)] |
| PTSD (n=342) | 1.7 (1.1-2.6) *  [2.8 (1.4)] | 1.4 (0.6-3.3) | 1.3 (0.8-2.0) | 2.3 (0.8-7.0) | 0.8 (0.4-1.7) | 0.8 (0.4-1.3) | 0.8 (0.4-2.0) |

Level of significance: ***p < 0.001, **p < 0.01, *p < 0.05. † Model adjusted for age group and gender only. ‡ Model adjusted for age group, gender, experience of financial hardship during childhood, current financial strain, and other types of CM (CPA, CEA, neglect and EDV). Among the 8,503 participants, 67 refused to report the perpetrator type, while 614 participants experienced CSA involving two or more perpetrator classes (these were excluded from the analysis above). CSA by unknown adolescents vs. CSA by known adolescent/ unknown adults were not reported in the pairwise comparison due to small sample size (n=51).

E‑value: minimum strength of association (risk‑ratio scale) that an unmeasured confounder would need to have with both the exposure and the outcome, conditional on measured covariates, to explain away the observed association; E‑value for the CI is the confounder strength required to move the confidence interval to include the null
